# Unveiling the Configurational Landscape of Carbamate: Paving the Way for Designing Functional Sequence-Defined Polymers

**DOI:** 10.1021/acs.jpca.3c02442

**Published:** 2023-08-25

**Authors:** Ariel F. Perez Mellor, Johanna Brazard, Sara Kozub, Thomas Bürgi, Roza Szweda, Takuji B. M. Adachi

**Affiliations:** † Department of Physical Chemistry, Sciences II, University of Geneva, 30, Quai Ernest Ansermet, Geneva 1211, Switzerland; ‡ Łukasiewicz Research Network − PORT Polish Center for Technology Development, Stabłowicka 147, Wrocław 54-066, Poland

## Abstract

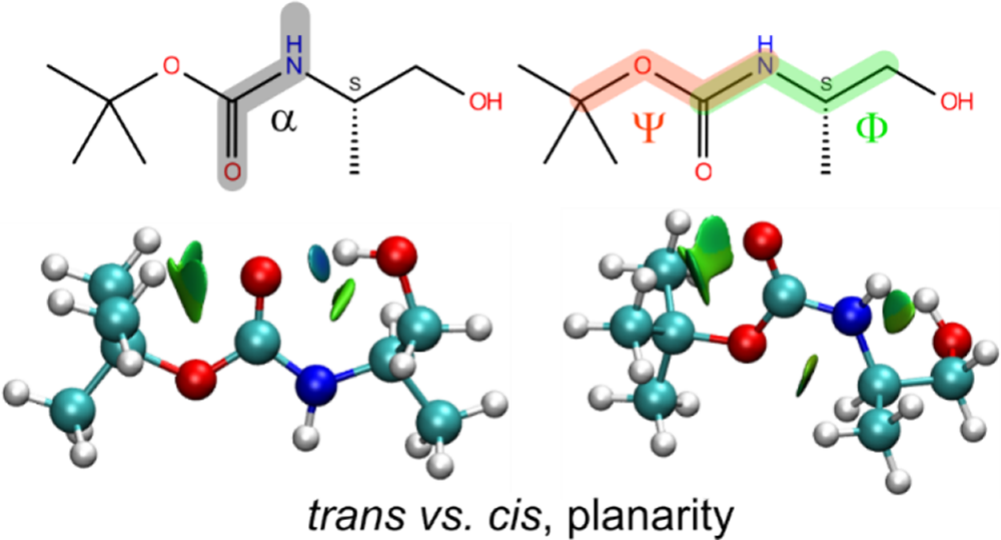

Carbamate is an emerging class of a polymer backbone for constructing sequence-defined, abiotic polymers. It is expected that new functional materials can be *de novo* designed by controlling the primary polycarbamate sequence. While amino acids have been actively studied as building blocks for protein folding and peptide self-assembly, carbamates have not been widely investigated from this perspective. Here, we combined infrared (IR), vibrational circular dichroism (VCD), and nuclear magnetic resonance (NMR) spectroscopy with density functional theory (DFT) calculations to understand the conformation of carbamate monomer units in a nonpolar, aprotic environment (chloroform). Compared with amino acid building blocks, carbamates are more rigid, presumably due to the extended delocalization of π-electrons on the backbones. *Cis* configurations of the amide bond can be energetically stable in carbamates, whereas peptides often assume *trans* configurations at low energies. This study lays an essential foundation for future developments of carbamate-based sequence-defined polymer material design.

## Introduction

The design and development of functional nanomaterials based on the control of structure–property relationships have been a valuable research strategy to address various societal challenges. In nature, complex and sophisticated structures are commonly formed using 20 canonical amino acids as building blocks. The primary amino acid sequence determines how a protein folds into the desired structure to perform its function. The protein folding and hierarchical self-assembly of biopolymers are achieved through the precise control of primary sequences.^1,2^ Therefore, significant efforts have been devoted to understand how the structures are controlled in nature and to achieve *de novo* design of new functional materials through the primary sequence control.^3,4^ With the development of sequence-defined peptide synthesis methods,^5−9^ the field of peptide- and protein-based functional nanomaterials has flourished to obtain sophisticated structures and fine-tuning functions.^1,10−12^

Although polypeptides are a promising class of materials, several challenges remain. For example, it takes time and cost to synthesize polypeptides of a defined sequence and the amount that can be synthesized is limited.^13^ Increasing the quantity of synthesized materials while reducing the cost of compounds is a key issue for applications. Material biodegradation is another aspect that needs to be addressed.^14^ As an alternative approach to overcome these problems, sequence-defined synthetic polymers (e.g., polypeptoids, oligocarbamates, oligo(triazol amide)s, polyesters, poly(phosphodiester)s, etc.) recently started to attract attention.^15−21^ The versatility of monomer structures, the chemical stability, and the tunability of properties in various solvents and environments are the advantages of abiotic polymers.^22^

Sequence-defined polycarbamates, known as polyurethanes, are a promising structural platform to be explored.^23^ One of the significant advantages of oligocarbamate materials is their easy fabrication at a large scale by one-pot, multistep synthesis as recently demonstrated.^24^ The precise monomer sequence control enables fine-tuning of the material properties.^25^ For example, it was reported how the insertion of carbamate links to the oligourea structure affects the shape of the oligomer.^26,27^ Therefore, they have been used as materials of sophisticated functionalities such as data storage materials,^28,29^ taggants in security technologies,^30−32^ and molecular transporters.^33,34^ As the promising aspects of sequence-defined oligo- and polycarbamates start to gain attention, it is essential to understand the configurational landscape of carbamate units compared with widely studied peptide backbones as a foundation for the further investigation of their single-chain folding and hierarchical self-assembly.

**Figure 1:**
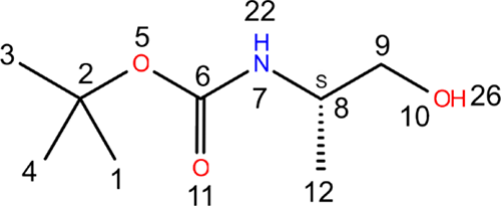
2D-molecular drawing and labeling of atoms. Carbamate can be regarded as a hybrid of amide and ester bonds.

In this article, we study the conformation of carbamate monomers (Boc-2-amino-1-propanol), a building block for sequence-defined polymers, in a nonpolar, aprotic environment (chloroform) by combining infrared (IR), vibrational circular dichroism (VCD), and nuclear magnetic resonance (NMR) spectroscopy with density functional theory (DFT) simulation. This approach has been shown to be powerful to investigate conformational landscape of various chemical systems.^35−47^ We applied it to establish a fundamental understanding of carbamate structure and how it differs from widely studied peptide units. The Boc group-protected carbamate monomer, see Figure 1, was chosen as a model system since this type of building block is used as an initiator unit for the iterative synthesis of sequence-defined polymer, which involves repetitive activation of OH terminal group by *N*,*N*′-disuccinimidyl carbonate, followed by chemoselective coupling of an amino alcohol monomer.^24^ Furthermore, we explored the role of the basis sets in spectral simulation to demonstrate that small basis sets can be used in larger systems (e.g., oligo- and polycarbamates) in the future. This work lays an essential foundation for studying a family of sequence-defined polycarbamates that are becoming an important class of materials with emerging applications.

**Figure 2:**
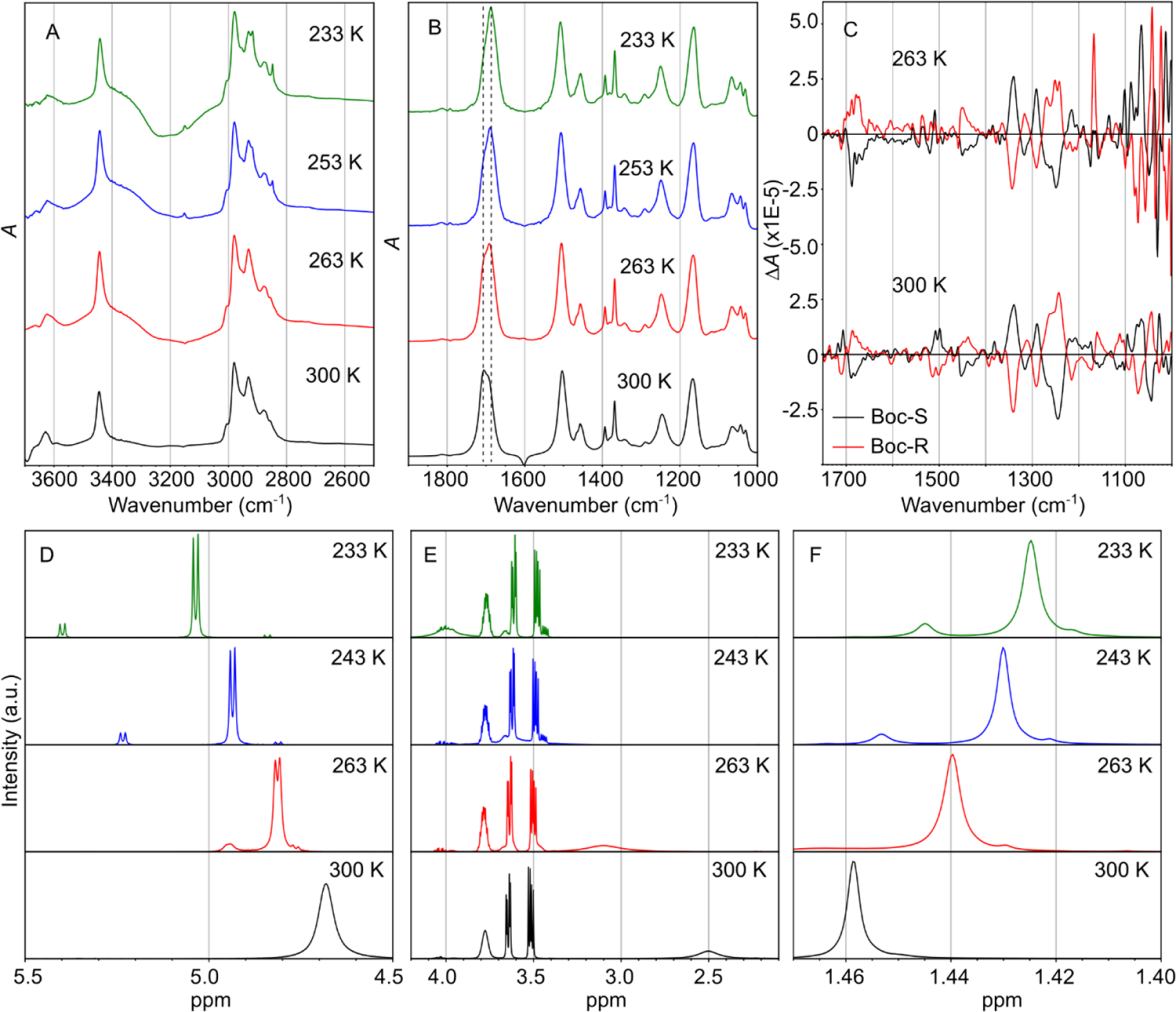
(A, B) IR spectrum, (C) VCD spectrum, and (D–F) NMR spectrum of the Boc-carbamate monomer at room and low temperatures.

**Figure 3:**
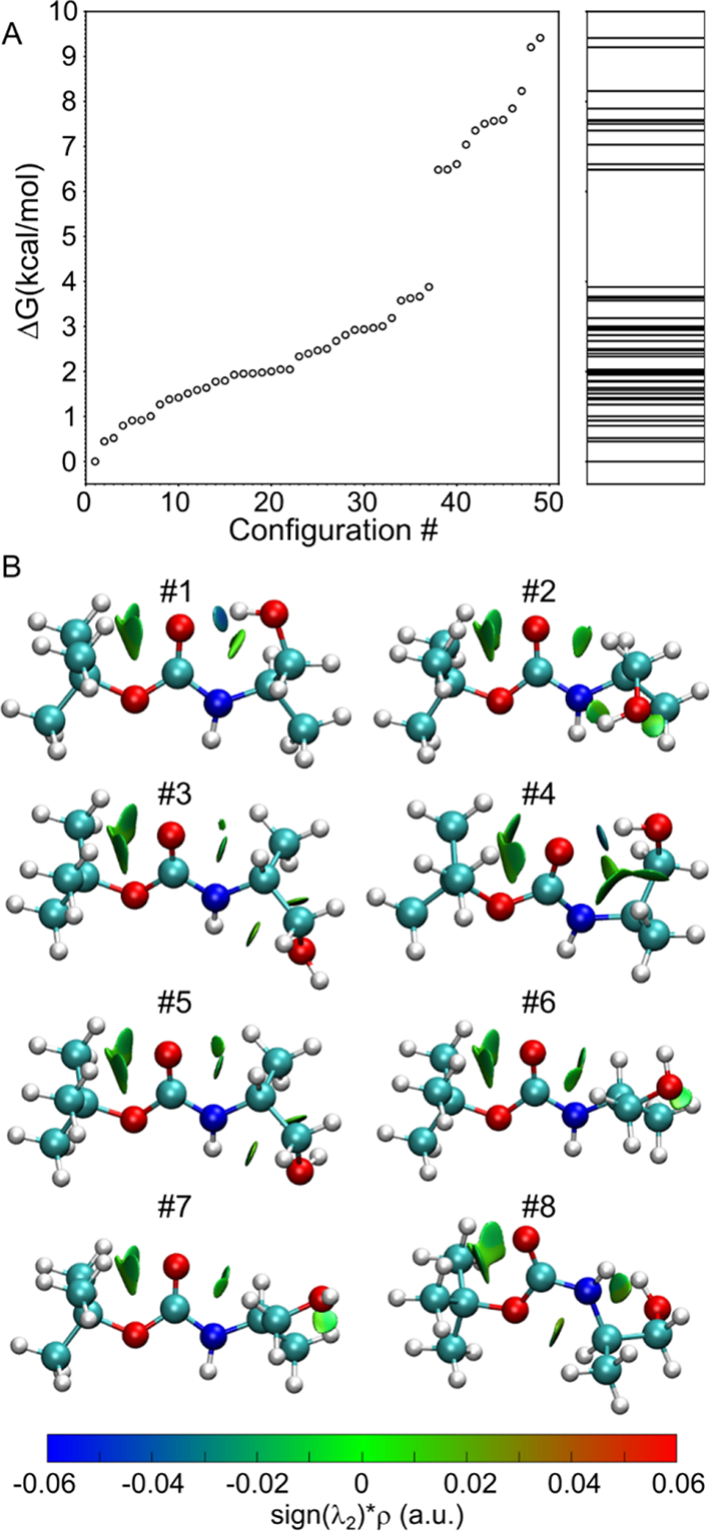
(A) Relative Gibbs free energy landscape of optimized geometries via HLT. The conformations were sorted in ascending order based on their energy and labeled for convenience. (B) NCI isosurface (s = 0.5) of the eight lowest energy conformers.

**Figure 4:**
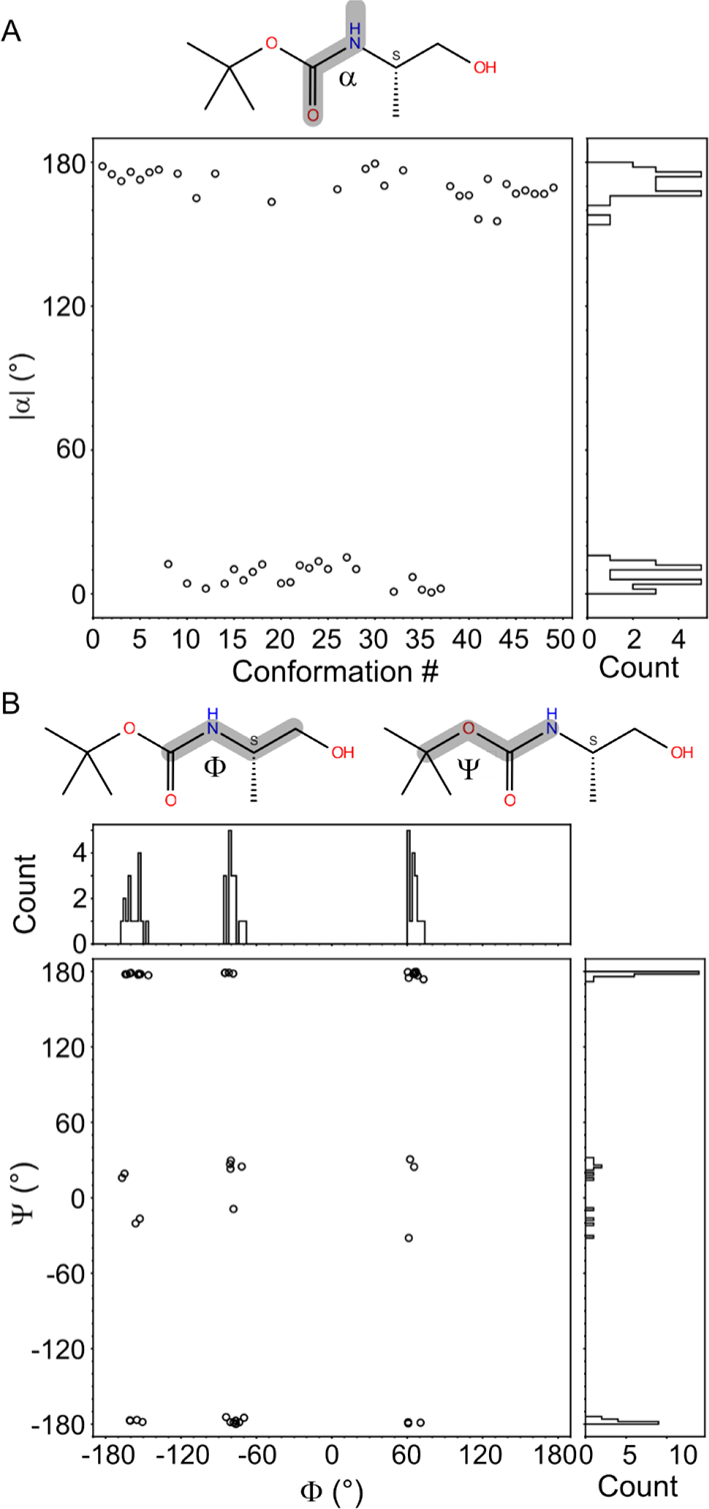
(A) Dihedral angle α measured for each configuration and its histogram. (B) The Ramachandran plot for the dihedral angle Φ and Ψ along with their histograms calculated from Boc-S.

**Figure 5:**
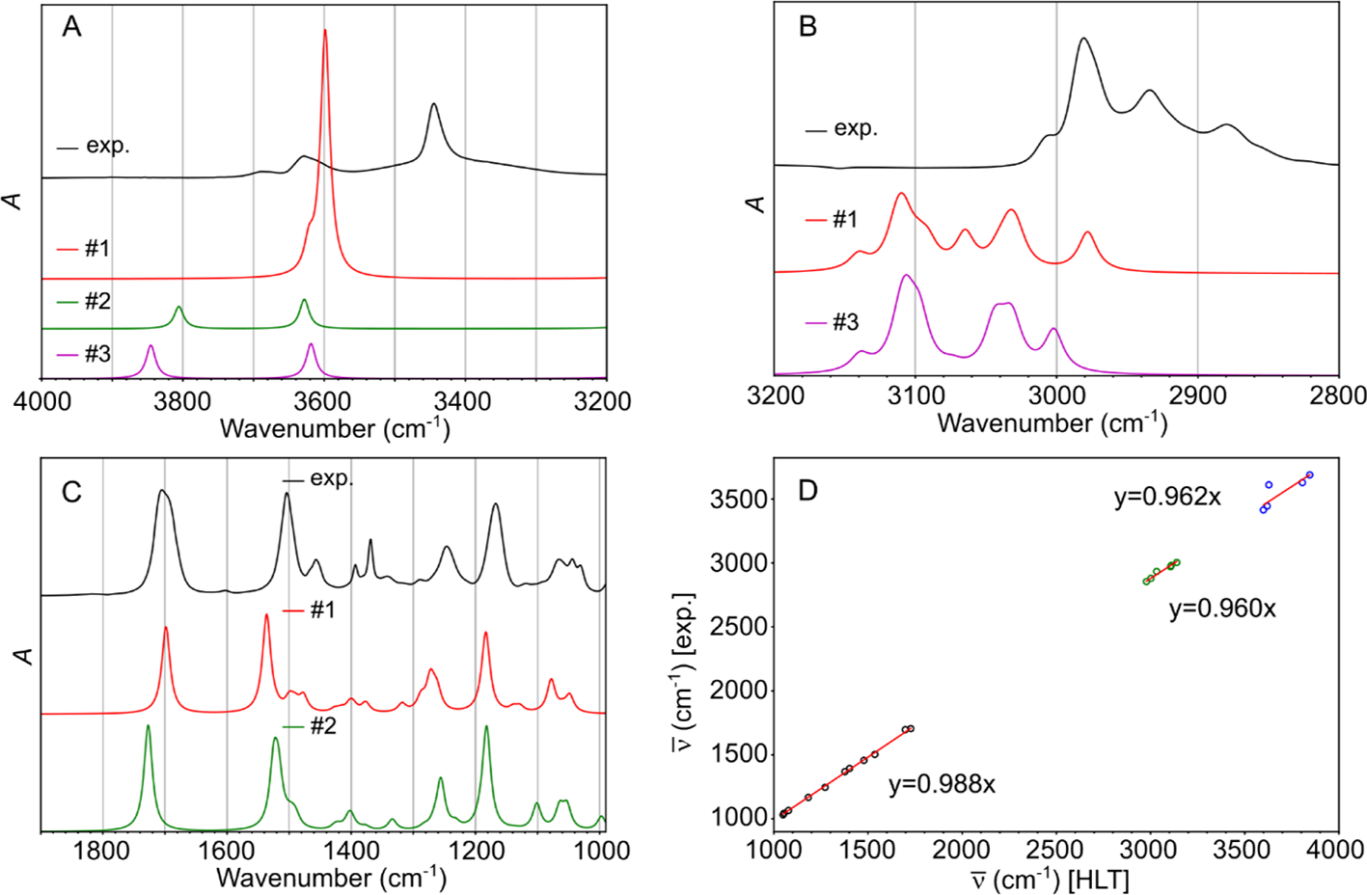
(A–C) Comparison of experimental (black) and simulated IR spectrum (the conformer #1: red, #2: green, #3: magenta) over three different wavenumber regions. (D) Vibrational peak positions from experiments and simulation plotted against each other. Using the shift between the two, scaling factor was obtained as a slope.

**Figure 6:**
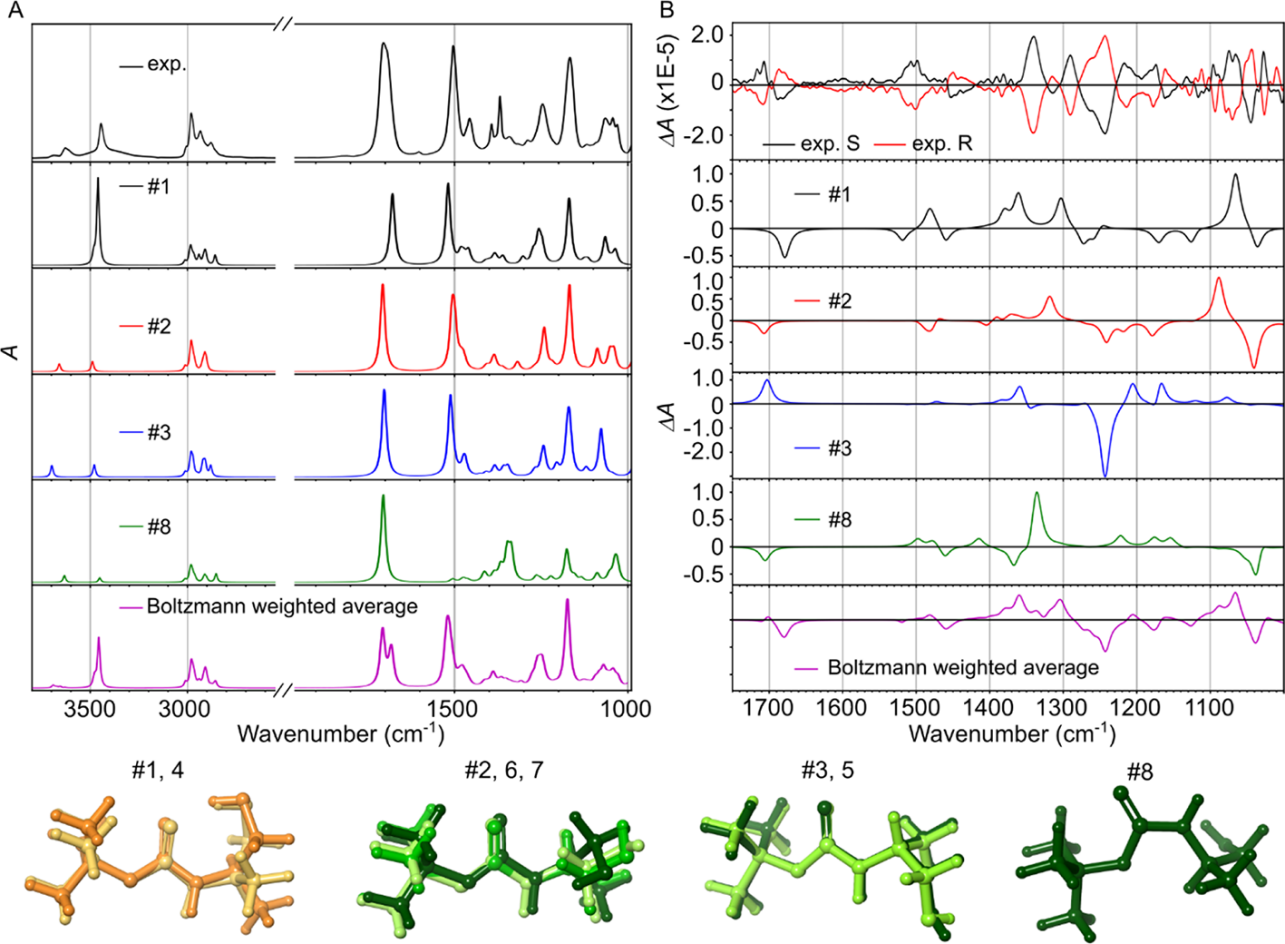
Comparison between experiments and simulation of (A) IR and (B) VCD spectrum. IR and VCD spectra are measured at the room temperature. The VCD spectra were calculated using the S enantiomer. The simulated IR and VCD spectra were scaled based on the factor obtained in Figure 5D.

## Experimental Methods

### Synthesis of Boc-2-amino-1-propanol (“Boc-carbamate”)

Chemoselective protection of amino alcohols with Boc-group was performed similarly to the reported procedure.^48^ (*S*)-2-amino-1-propanol (Apollo Scientific 98%, *ee*: 97%, 1.04 mL, 1 equiv, mass) was dissolved in water (MiliQ, 3 mL) and (Boc)_2_O (Angene 95%, 3.37 mL, 1.1 equiv) was added dropwise. The reaction mixture was stirred overnight at room temperature. Afterward, the product was isolated by extraction with ethyl acetate (Stanlab) and dried under vacuum. The resulting product in the form of white powder was obtained with 85% yield. The chemical structure of the product was confirmed via ^1^H NMR analysis. The same procedure was used to synthesize Boc-(*R*)-2-amino-1-propanol but this time using (*R*)-2-amino-1-propanol as the reagent.

### IR and VCD Spectroscopy

IR/VCD spectra (1000–1800 cm^–1^ region) of the Boc-carbamate monomer (both enantiomers) in solution were recorded using an FTIR spectrometer (Tensor 27 Bruker) equipped with a VCD module (PMA 50 Bruker). The handedness of circularly polarized light was modulated using a Hinds PEM 90 photoelastic modulator (PEM), which was set to a λ/4 retardation with a central frequency of 1400 cm^–1^. A lock-in amplifier (SR830 DSP) was employed for demodulation. An optical low-pass filter (800–2000 cm^–1^) was placed before the PEM to improve the signal-to-noise ratio. Two temperatures, 300 and 263 K, were examined. A variable temperature cell from Specac was utilized to achieve the desired temperatures, with liquid nitrogen serving as the refrigerant. Each spectrum was collected over 8 h (34,080 scans), with a spectral resolution of 4 cm^–1^. Deuterated chloroform (99.80 %D, Eurisotop) was used as the solvent to prepare a concentration of 100 mmol L^–1^ of Boc-carbamate monomer. Half of the sum of the VCD spectra of the two enantiomers was considered as the baseline (see Figure S1). Additionally, IR spectra in the range of 1000–3600 cm^–1^ were recorded with the same setup, using a spectral resolution of 2 cm^–1^ and 100 scans. In this case, the optical filter was replaced by a different one (800–4000 cm^–1^) and four temperatures, 300, 263, 253, and 233 K, were evaluated. The background for this measurement was the IR spectrum of the pure solvent at each temperature. All measurements were performed with a 200 μm path-length cell sealed with CaF_2_ windows.

### NMR Spectroscopy

^1^H NMR spectra of both Boc-2-Amino-1-propanol enantiomers were recorded in deuterated chloroform (99.8 atom %D, Sigma Aldrich) using an Avance III HD 600 MHZ NMR spectrometer (BRUKER) equipped with probes: BBI and BBO. Analyses were performed at the same temperature range as IR/VCD spectroscopy. The recorded spectra were calibrated according to the chloroform signal (7.26 ppm for all temperatures) and evaluated in MNova software.

## Theoretical Methods

**Scheme 1:**
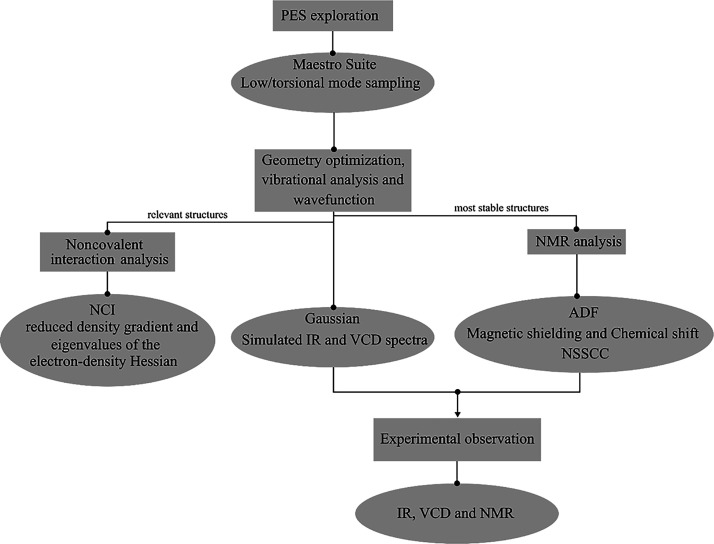
Workflow That Highlights the Methodology Followed in This Research

We employed a comprehensive methodology depicted in Scheme 1. Briefly, we generated the conformations of the Boc-carbamate monomer in chloroform using a conformational search method that is described in detail below. The resulting geometries were optimized and used to simulate IR, VCD, and NMR spectra. We then compared these simulated spectra to experimental spectra to validate the accuracy of the generated conformations and to perform a detailed analysis of their characteristics. The details of each step are described in the following.

### Exploration of the Potential Energy Surface (PES)

The conformational landscape of the Boc-carbamate monomer was thoroughly explored using the conformational search tool implemented in the Maestro program.^49^ A mixed torsional/low-mode sampling method in chloroform was employed. Solvation effects were simulated using the Generalized-Born/Surface-Area (GB/SA) analytical model. The interaction between the atoms in the molecule was described by the force field OPLS-2005.^50^ Then, the potential energy was minimized by the Polak-Ribière Conjugate Gradient (PRCG) method. The initial geometry was obtained after a prior optimization of a drawing of the molecule based on chemical intuition. Finally, an energy window of 10.02 kcal mol^–1^ was applied for saving structures. As a result, 49 nonsuperimposable mirror image conformations were generated. This approach has proven particularly useful for peptides, as their behavior can be complex and influenced by factors such as intramolecular hydrogen bonding, steric hindrance, and solvent effects. Previously, it has been effectively employed in medium-sized linear^41^ and cyclic peptides.^43^

### Geometry Optimization and Vibrational Analysis

Geometry optimization and normal mode analysis for the 49 generated conformations were carried out within the framework of DFT^51,52^ with the help of Gaussian 16 software.^53^ For this purpose, the hybrid functional B3LYP^54−56^ along with two of Pople’s basis sets, 6-31g(d,p) and 6-311g++(d,p)^53,57^ were used. Empirical D3 dispersion corrections^58^ were also applied. The effects of solvation, with chloroform as the solvent, were taken into account by placing the molecule in a cavity within the reaction field of the solvent using the Polarizable Continuum Model (PCM).^59^ In what follows, we will refer to the levels of theory as the low-level theory (LLT) and high-level theory (HLT). The LLT encompasses B3LYP-D3/6-31g(d,p)/PCM, while the HLT comprises B3LYP-D3/6-311g++(d,p)/PCM. The two levels were applied in parallel to assess the performance of the LLT needed for the investigation of larger systems, such as oligocarbamates. IR and VCD spectra at 0 K were obtained after the convolution of the computed transition dipole moment and the rotatory strength at a given frequency using a Lorentzian function of a 10 cm^–1^ full-width half-maximum (FWHM). To account for the incompleteness of the basis set, anharmonic and solvation effects, two sets of scaling factors were used depending on the level of theory. They were obtained based on the experimental IR absorption spectra (see the section for the Figure 6). Prior to this study, both levels of theory have been effectively used to simulate the IR and VCD spectra of cyclo dipeptides and their dimers.^60^

### Simulation of ^1^H NMR Spectra

The ^1^H spectra at 0 K of the conformers were simulated using the Amsterdam Modeling Suite (AMS) software.^61,62^ First, the most stable geometries found by using LLT and HLT were reoptimized at the DFT level using the hybrid functional PBE0^63^ with the TZP basis set.^64^ Subsequently, Single-point calculations were conducted to compute the magnetic shieldings and chemical shifts. In this case, the OPBE functional together with the TZ2P-J basis set was employed. The reason the optimization of the geometries was not done directly by OPBE/TZ2P-J was to reduce the cost of the computation. This methodology has proven to be robust for the prediction of nuclear magnetic constants.^65^ Although we chose to use a well-established methodology that provides a balance between accuracy and computational cost with the idea of scaling to oligocarbamates in the future, we do not imply that using B3LYP in place of PBE0 would be ineffective. We avoided it because it requires us to conduct a benchmark comparison between the two to use B3LYP, which falls outside the scope of this article. Finally, the nuclear spin–spin coupling constant (NSSCC) was calculated through the CLP routine^66,67^ included in AMS. The frequency of the applied magnetic field was 400 MHz, while the FWHM was set at 0.02 ppm. The threshold values for the chemical equivalences, magnetic equivalences, and strong/weak coupling were 0.00001, 0.01, and 0.5, respectively. All the computed chemical shifts are relative to the ^1^H shifts of tetramethylsilane (TMS), which was also calculated using the same protocol (Figure S2A). As a control test, the ^1^H NMR spectra of chloroform were simulated (Figure S2B). A theoretical chemical shift of 7.59 ppm for ^1^H is in a good agreement with the experimental values of 7.26 ppm.

### Analysis and Visualization of the Noncovalent Interactions

Intramolecular interactions were visualized and analyzed using the Non-Covalent Interaction (NCI) technique.^74^ A detailed description of this method and its applicability can be found elsewhere.^68^ In short, the NCI technique is a topological analysis method that evaluates the electron density ρ and its reduced gradient s(ρ) in the regions of weak electron densities and small-reduced gradients. The electron density was obtained through self-consistent field (SCF) wavefunction printed after geometry optimization at HLT. To visualize the reduced gradient, we plotted isosurfaces of s(ρ) using an RGB color map based on the sign of the second eigenvalue (λ_2_) of the Hessian matrix multiplied by the ρ value. Reddish isosurfaces indicate repulsive regions (λ_2_ >0), bluish isosurfaces represent favorable interactions (λ_2_ <0), and greenish isosurfaces correspond to weak delocalized interactions (λ_2_ ∼0). VMD software^69^ was used for visualization.

## Results and Discussion

Figure 2 summarizes the experimental IR, VCD, and NMR spectra of the Boc-carbamate monomer obtained at various temperatures. To enhance the clarity of the text, the IR spectra can be categorized into two distinct regions: the high-frequency region (HFR) and the low-frequency region (LFR). The HFR, which spans from 2800 to 4000 cm^–1^, encompasses the −XH stretching vibrations (νXH), where X can represent oxygen (O), nitrogen (N), or carbon (C) atoms. In contrast, the LFR comprises two subregions: the IR fingerprint region (1000–1500 cm^–1^) and the —C=O stretching vibration region (1500–1800 cm^–1^).

The positions of νOH and νNH vibrational frequencies deliver critical information concerning the conformation and presence of intermolecular or intramolecular hydrogen bonds (HBs) due to their sensitivity to subtle structural modifications (see Figure 2A). To eliminate the potential influence of intermolecular HB formation resulting from aggregation, we executed a concentration-dependent analysis of the IR spectra (refer to Figure S3). The analysis indicates that the IR spectra exhibit no substantial changes other than variations in intensity. Based on these findings, we conclude that, within the tested concentration range, aggregation effects are negligible, and the possible HBs observed can be characterized as intramolecular.

At room temperature, an intense peak arises at 3445 cm^–1^ due to the νNH vibration. It should be noted that an intramolecular HB involving the NH group is impossible in this small system. Therefore, this peak considers the free νNH vibration. Furthermore, two peaks are observed around 3588 and 3626 cm^–1^, which can be assigned to the vibration νOH without HBs and embedded in two different environments. The broadband below the νNH peak is due to νOH being hydrogen-bonded (the detailed assignment can be found in the later section). As the temperature decreases, this band becomes more pronounced, indicating that the structures with the OH group forming HBs became more stable and more populated. So far, three different environments for the νOH have been identified, suggesting at least three different groups of conformations. The remaining peaks in the HFR correspond to aliphatic groups. Unfortunately, they are highly congested, and the extraction of information is often challenging due to lower sensitivity to conformational changes.

The LFR (Figure 2B) exhibits several well-defined peaks, with the most intense at approximately 1166, 1503, and 1706 cm^–1^ at room temperature (300 K). The peak at 1706 cm^–1^ is typical of the stretching motion of the carbonyl group, and a closer examination reveals a shoulder at 1690 cm^–1^ (black dashed lines). Since the molecule only contains one carbonyl group, the presence of the shoulder suggests the existence of at least two families of conformers with different environments around the carbonyl group. This interpretation is supported by the change in the profile of this band with temperature, with the intensity of the shoulder at 1690 cm^–1^ increasing at lower temperatures and becoming the main band at 233 K. This indicates that the family of conformers corresponding to the 1690 cm^–1^ band is the most stable and therefore the most populated at lower temperatures. Combining this information with that previously extracted from the νOH region, we can deduce from an experimental standpoint that the most stable conformers exhibit an intramolecular HB directed toward the carbonyl group. This interaction results in a shift of the νCO vibrational band toward lower frequencies. The remaining two families related to the local environment of the νOH group share a similar position for the νCO band. In such cases, the carbonyl group remains free, undisturbed.

The VCD spectra of the two enantiomers at room temperature are illustrated in Figure 2C. The carbonyl stretching region is characterized by two bands with opposite signs, centered at 1687 and 1712 cm^–1^. These observations provide further evidence for the existence of two distinct local environments surrounding the carbonyl functional group. The one that corresponds to the intramolecular HB has a negative sign, while that of the undisturbed CO group has a globally positive band. All this description holds for the S enantiomer, and note that it is the opposite for the R enantiomer. Additionally, a notable band is observed in the amide II (βNH: bending mode) region at a wavenumber of 1500 cm^–1^. The VCD spectra also display significant activity at wavenumbers of 1340, 1291, and 1241 cm^–1^, further highlighting the complex vibrational behavior of the molecular system under investigation. To correctly assign the latter bands, quantum chemistry simulations are performed later in this article. Upon lowering the temperature to 263 K, distinct alterations manifest in the VCD spectra. The positive band, associated with conformations featuring an undisturbed CO group in the S system, is no longer present, while an increase in the absolute value of the negative band is observed. This data bolsters the hypothesis that a shift in population occurs from conformations containing the undisturbed CO group to those with a hydrogen-bonded CO group as the temperature decreases. Intriguingly, the vibrational activity within the amide II (βNH) region also ceases to be detectable. For the residual bands located above 1100 cm^–1^, there are no noteworthy deviation.

NMR spectroscopy analysis, as depicted in Figure 2D–F and the complete spectra in Figure S4, uncovers temperature-dependent variations in the distribution of diverse conformational ensembles. Four distinct regions can be clearly discerned, spanning from low to high magnetic fields. In the low-field region, the amide protons (4.5–5.0 ppm) are discernible, along with the alpha carbon protons (C^α^H) and methylene protons (CH_2_) within the range of 3.0–4.0 ppm. In the high-field region, the proton signals pertaining to the Boc group (1.40–1.47 ppm) and the methyl group protons of the residue (1.00–1.30 ppm) are observable. Interestingly, the signals demonstrate a narrowing of the peak widths across all regions when the temperature decreases. At room temperature, a single band appears in the amide proton region, likely due to the molecules transitioning between multiple conformations, rendering the NMR spectroscopy time scale insufficient to resolve the peaks of each conformer individually. Conversely, at lower temperatures, the molecules dwell long enough within each conformation for NMR spectroscopy to detect the peaks corresponding to distinct sets of conformers. At the lowest recorded temperature, three doublets emerge in the amide proton region, centered at 4.84, 5.03, and 5.40 ppm. This observation implies the presence of three unique local environments for the amide proton. A broadband exhibiting a considerable temperature-dependent shift, ranging from 2.35 ppm at room temperature to 4.00 ppm at 233 K is assigned as an OH proton. NH and OH proton peaks show downshift as lowering temperature, which is due to the deshielding that occurs in the hydrogen bond. OH is more polar than carbamate NH, and therefore, the temperature dependent peak shift is more significant. OH hydrogen bonds are stronger and have broader energy range reflected in bigger shift changes upon temperature. The complete assignment of the bands will be made in the following sections based on quantum chemistry calculations.

The experimental results provided qualitative evidence for several sets of conformers in solution. To gain quantitative information on the structural landscape of the Boc-carbamate monomer, we conducted a theoretical investigation. As described in the methods section, we used HLT to optimize the 49 conformations obtained during the PES exploration. Gibbs free energies relative to the lowest energy conformation values were then calculated at standard ambient pressure and temperature (298.15 K and 1 atm) and plotted in ascending order (Figure 3A). The energy gap between the lowest and second-lowest energy conformations (#1 and #2, respectively) is 0.45 kcal mol^–1^, and six conformations are within 1 kcal mol^–1^. Notably, conformer #38 is separated from conformer #39 by approximately 2 kcal mol^–1^, a point to which we shall return later.

Figure 3B depicts the NCI isosurfaces for the eight lowest conformers, while Figure S5 presents a 2D plot of *s* versus ρ. Table 1 provides an overview of the interactions present in the eight most stable conformers, along with the corresponding distances that characterize each interaction. These conformers are stabilized primarily by weak hydrogen bond (*w*HB) interactions between the *tert*-Butyl and carbonyl group of the backbone (2(-CH_3_)...O_11_=C_6_−), which are characterized by ρ ∼0.012 at the critical point (s ∼0). Notably, the most stable structure #1 and the structure #4 contain strong hydrogen bond (*s*HB) interactions between the hydroxy and carbonyl groups (−O_10_H...O_11_=C_6_−), described by ρ ∼0.035 and a short distance *d*(−CO...HO−) ∼1.84 Å. The remaining conformers exhibit other *w*HB interactions, such as HB between the hydroxy group and lone pair (lp) of the nitrogen (−O_10_H...(lp)–N_7_−) in the amide bond (#2 and #8) and HB between the hydrogen of the amide bond and the oxygen of the hydroxy group (#3 and #5) (−N_7_H...(lp)–O_10_−), all of which fall in the region ρ <0.012 at s ∼0. Remarkably, the structure #8, found at only 1.27 kcal mol^–1^ higher in energy than the global minimum, contains the amide bond in the *cis* configuration. In this specific case, there is HB formation between the alpha hydrogen and lone pair of the oxygen atom (−C^α^_8_H...(lp)–O_5_−) in the carbamate group, with a distance of *d*(−C^α^_8_H...–O_5_−) ∼2.33 Å and ρ = 0.016. It is worth noting that the stabilization of the *cis* form in oligopeptides is highly unlikely due to steric clashes between the groups of alpha carbon atoms. A more detailed discussion of *cis* configurations will be discussed further at the later section.

**Table 1:** Details of Noncovalent Interactions in the Eight Lowest Conformers (See Table S1 for the Additional Information of the Energy for the Eight Lowest Conformers)^a^

conformation	Δ*G* (kcal/mol, SATP)*	Interaction	ρ^**^ (a.u)	distance^***^ (Å)	distance for charge delocalization^****^ (Å)
#1	0.0	–O_10_H...O_11_=C_6_—	0.033	1.84	1.34, 1.35, 1.23
		2(−CH_3_)...O_11_=C_6_—	0.012	2.45	
#2	0.45	2(−CH_3_)...O_11_=C_6_—	0.012	2.45	1.35, 1.36, 1.22
		–C^α^_8_H...O_11_=C_6_—	0.010	2.58	
		–O_10_H...(lp)–N_7_–	0.010	2.60	
		–C_12_H_3_...(lp)–O_10_–	0.009	2.71	
		–C_9_H_2_...O_11_=C_6_—	0.007	2.76	
#3	0.52	2(−CH_3_)...O_11_=C_6_—	0.012	2.45	1.35, 1.36, 1.22
		–N_7_H...(lp)–O_10_–	0.010	2.47	
		–C^α^_8_H...O_11_=C_6_—	0.010	2.51	
		–C_12_H_3_...(lp)–O_10_–	0.008	2.65	
		–C_12_H_3_...O_11_=C_6_—	0.004	3.07	
#4	0.80	–O_10_H...O_11_=C_6_–	0.034	1.82	1.35, 1.36, 1.23
		2(−CH_3_)...O_11_=C_6_–	0.012	2.44	
		–C_12_H_3_...O_11_=C_6_–	0.007	2.80	
		–C_12_H_3_...(lp)–O_10_–	0.010	2.70	
#5	0.91	2(−CH_3_)...O_11_=C_6_–	0.012	2.44	1.35, 1.36, 1.22
		–N_7_H...(lp)–O_10_–	0.008	2.49	
		–C^α^_8_H...O_11_=C_6_–	0.008	2.56	
		–C_12_H_3_...O_11_=C_6_–	0.005	2.93	
#6	0.92	2(−CH_3_)...O_11_=C_6_–	0.012	2.45	1.35, 1.36, 1.22
		–C^α^_8_H...O_11_=C_6_–	0.009	2.53	
		–C_12_H_3_...(lp)–O_10_–	0.009	2.66	
		–C_9_H_2_...O_11_=C_6_–	0.006	2.89	
#7	1.0	2(−CH_3_)...O_11_=C_6_–	0.012	2.45	1.35, 1.36, 1.22
		–C^α^_8_H...O_11_=C_6_–	0.010	2.52	
		–C_12_H_3_...(lp)–O_10_–	0.009	2.58	
		–C_9_H_2_...O_11_=C_6_–	0.005	2.94	
#8	1.3	–C^α^_8_H...(lp)–O_5_–	0.016	2.32	1.34, 1.37, 1.22
		–O_10_H...(lp)–N_7_–	0.014	2.42	
		2(−CH_3_)...O_11_=C_6_—	0.012	2.45	

a *Relative Gibbs free energy calculated at standard ambient pressure and temperature (1 atm and 298.15 K), **the electron density at the critical point, ***the minimum distances between hydrogen and functional group, and **** *d*(O_5_-C_6_), *d*(N_7_-C_6_), and *d*(O_11_-C_6_), respectively.

The occurrence of *s*HB formation was also explored by measuring the distance between the H atom of the hydroxy group (atom labeled as H_26_) and all other atoms in all 49 conformers (Figure S6). As expected from chemical intuition, two distinct sets of *s*HB interactions can be distinguished. The first set involves the formation of *s*HB with O_11_, which is observed in the most stable conformer. The *s*HB in the most stable conformer contributes to the stabilization of the conformations and reduces the total energy of the system, as evidenced by the 0.45 kcal mol^–1^ energy difference between conformers #1 and #2. The second set involves sHB formation with O_5_, with a distance of *d*(−CO–...HO−) ∼1.84 Å, as observed in structure #12 (1.6 kcal mol^–1^), and helps to stabilize the *cis* geometry of the amide bond. This type of interaction is not usually observed in oligopeptides.

The quantification of *cis*/*trans* isomerization was accomplished through dihedral angle analysis. The dihedral angle surrounding the amide bond is denoted as α(O_11_C_6_N_7_H_22_), as illustrated in the inset of Figure 4A. The measurement of angle α was performed across 49 conformations, and its distribution is depicted in Figure 4A. Notably, the results indicate that nearly half (21 out of 49) of the conformers with energy levels below 10 kcal mol^–1^ exhibited *cis* configurations, representing a remarkable deviation from the established literature on peptides. This divergence is attributed to the stabilization of the *cis* structures, which is mainly facilitated by −C^α^_8_H...(lp)–O_5_– (e.g., conformer #8) or −CO–...HO– (e.g., conformer #12) interactions. Further investigations are underway to elucidate this finding and gain a deeper understanding of the distinct characteristics of oligocarbamates versus oligopeptides.

A Ramachandran plot (Figure 4B) was created to gain deeper insight into the role of dihedral angles in stabilizing the optimized geometries. The angle Ψ(C_2_O_5_C_6_N_7_) was observed to consistently exhibit only three values: −180°, 0°, or 180°. Those conformations with Ψ ∼0 were found to be highly energetic and are responsible for the ∼2 kcal mol^–1^ energy gap observed in Figure 3A. The structures #37 and #38 that give rise to this energy gap are depicted in Figure S7. The planarity of Ψ combined with the two planar positions of the adjacent amide bond (*cis*/*trans*) leads to the consequence that the backbone of the Boc-carbamate monomer consistently adopts a planar conformation, as previously reported.^70^ This suggests that the O_5_, C_6_, O_11_, N_7_, and H_22_ atoms are always in one plane. To further support this conclusion, molecular orbitals calculations were performed on the selected structures (Figure S8). The results show the delocalization of the highest occupied molecular orbital (HOMO), HOMO-1, HOMO-2, and HOMO-3 along O_5_ and O_11_ indicating deconjugation of the heteroatom (−σ bond)-carbon (−π bond)-heteroatom system. This restriction of rotation around the formal single σ bond is also supported by the atomic charges computed from the atomic axial polar tensor (APT) of the eight most stable structures (Figure S9). In this respect, the backbone of the carbamate monomers can be seen as a center of positive charge (C_6_) surrounded by three negative charges (O_5_, O_11_, and N_7_), with distances between these atoms summarized in Table 1. The similar distances *d*(O_5_–C_6_) and *d*(C_6_–N_7_) further highlights the pseudo-double bond character of the carbamate motif.

The angle Φ(C_6_N_7_C_8_C_9_) is found to be populated around −180°, −60°, and 60°, rather than being randomly distributed. This stabilization of the angle is primarily driven by the steric interaction between the methyl group at the chiral center and the backbone, as well as hydrogen bonding between O–H...O in some conformers. The results for the S enantiomer are presented in Figure 4, and the sign of the Φ angle would flip if the R enantiomer were used instead. No conformers with Φ = 180° were observed for the S enantiomer, but some were present for the R enantiomer. Conversely, there were no conformers with Φ = −180° for the R enantiomer. This finding suggests that control over the torsion angle may be achievable through manipulation of the enantiomers.

A set of conformers depicted in Figure 3B was used to interpret the experimental IR, VCD, and NMR spectra. The comparison between the simulated (at HLT) and experimental IR spectra at room temperature was used to probe the presence of specific Boc-carbamate conformers in experiments. The detailed assignment of the vibration modes was also performed (Table 2). The solid black curves in Figure 5A–C represent the experimentally obtained IR spectrum of Boc-carbamate in chloroform in three spectral regions. The experimental spectrum was initially compared to the spectrum of the lowest energy conformer (#1), as depicted by the red solid curves in Figure 5A–C. Despite a good level of agreement between the experiment and spectrum of #1, not all the experimental features can be fully explained, particularly the number of bands in the OH stretch region (3200–4000 cm^–1^) and the shoulder in the CO stretch region (∼1690 cm^–1^). These discrepancies highlight the need to consider the presence of higher energy conformers under the experimental conditions, as previously discussed. The spectra of the second and third lowest energy conformers (#2 and #3) were added to the comparison, as shown in Figure 5A–C. Based on the full comparison between the experiment and simulation (Table 2), a correlation plot was established and a linear fit was used to obtain the scaling factor to shift the simulation results toward the experimental ones (Figure 5D). The scaling factor was established separately for each of the three spectral regions. The correction factor arises from the incompleteness of the basis set, anharmonic and solvation effects. The scaling factor determined here can be used in future oligocarbamate studies. This will ensure consistency and accuracy in interpreting the IR and VCD spectra for these more complex systems.

**Table 2:** Assignment of Experimental IR/VCD Bands^a^

ν̅_exp_ (cm^–1^)	ν̅_sim_ (scaled, cm^–1^)	ν̅_sim_ (cm^–1^)	Int. (IR, KM/Mole)	Int. (VCD, 10^–44^ esu^2^ cm^2^)	assignment	type*	conformer #
3689	3697	3845	72		νOH env_1_	A	3
3629	3660	3806			νOH env_2_	B	2
3445	3483	3622	63		νNH	C	1
	3488	3628					2
	3480	3619	75				3
3415	3461	3599	578		νOH_bound_	D	1
3004	3014	3140	25		νCH_3_(Boc)	E	1
	3014	3139	25				2
	3014	3139	22				3
2981	2987	3111	61		νCH_3_(Boc)	F	1
	2985	3110	59				2
	2984	3109	65				3
2934	2908	3029	45		νC^α^H + νCH_3_(res)	G	1
	2924	3046	28				2
	2950	3073	8				3
2855	2859	2978	68		νCH_2_	H	1
	2904	3025	41				2
	2882	3002	65				3
1705	1706	1727	502	–24	νCO + βNH	I	2
	1702	1723	534	64			3
1697	1678	1698	476	–45			1
1504	1518	1536	537	–21	βNH	J	1
	1506	1525	292	1			2
	1511	1529	480	–2			3
1467	1481	1499	36	39	βCH_3_(res) + βCH_2_ + βOH	K	1
	1478	1496	42	–19	βCH_3_(res) + βNH		2
	1473	1491	46	6			3
1457	1459	1477	86	–26	βOH + wCH_2_	L	1
1393	1380	1396	27	28	wC^α^H + wCH_2_ + βOH	M	1
1369	1360	1377	55	64	ρC^α^H + wCH_2_ + βOH	N	1
-	1336	1352	268	231	wC^α^H + βOH + νCN	O	8
1289	1302	1318	47	62	deloc. Mode	P	1
1246	1272	1288	72	–32	deloc. Mode	Q	1
	1257	1273	170	–21	deloc. Mode	R	1
1167	1169	1183	438	–27	collective mode (Boc)	S	1
	1168	1182	486	–7			2
	1167	1181	241	123			3
1065	1065	1078	179	138	deloc. Mode	T	1
1031	1036	1048	92	–55	deloc. Mode	U	1

a The mode type (*) refers to the normal mode displacement illustrated in Figure S10. The main characteristic movement is represented by lowercase letters: ν, stretching; β, bending; w, wagging; ρ, rocking. The delocalized modes are represented by deloc. mode.

After the correction based on the scaling factor, the experimental IR and VCD bands are properly assigned. The bands at 3689 and 3629 cm^–1^ are attributed to the νOH stretching vibrations in two distinct environments. The former corresponds to conformer #3 (−N_7_H...(lp)–O_10_−), while the latter is associated with conformer #2 (−O_10_H...(lp)–N_7_−). The band at 3445 cm^–1^ is assigned to the νNH stretching vibrations of the three most stable conformers, which are not sensitive to conformational changes in the IR spectra (3488, 3483, and 3480 cm^–1^). The broad band centered at 3415 cm^–1^ is ascribed to the νOH stretching vibrations of the hydroxyl group bound to the carbonyl group (−O_10_H...O_11_=C_6_—), as observed in the global energy minimum conformation. A complete assignment of the stretching region for aliphatic groups is presented in Table 2. In the νCO region, two bands can be attributed to the νCO vibrations coupled with βNH (mode type I) for two distinct groups of conformers. The high-energy peak at 1705 cm^–1^ is associated with conformers #2 and #3, whereas the lower-energy peak at 1697 cm^–1^ corresponds to conformer #1. This assignment aligns with the observed shift in population, favoring the most stable conformation as the temperature decreases. The band at 1504 cm^–1^ is assigned to the βNH (type G) of the three most stable structures.

In Figure 6, we compare the experimental and simulated spectra (IR, VCD) for two enantiomers of Boc-carbamates. As expected, the IR spectra of the two enantiomers are identical within the experimental resolution (Figure S11), while their VCD spectrum shows a mirror image (Figure 6B, top). Although matching the experiments and simulations for a system with multiple conformers is challenging, our results show a good agreement. The simulated IR and VCD spectra for each group of conformers are presented in Figure 6A,B along with the experimental spectrum measured at room temperature (top). The IR and VCD spectrum from the Boltzmann weighted averaging of conformers (at room temperature) was also plotted (Figure 6A,B, bottom). However, we treat the Boltzmann weighted spectrum as qualitative, because the energy difference between the five conformers (#2 to #7) is within 0.5 kcal/mol that is below the limit of the chemical accuracy of the calculations. The challenge of quantitatively compare experimental and simulated spectra has been highlighted in a recent publication.^71^ A new approach to consider the weight of each conformation more accurately despite the uncertainty of the calculated energy has been proposed^71^ and this could be implemented to our study in the future. The VCD spectra are particularly sensitive to a conformational variation, particularly around 1690 and 1706 cm^–1^ (C=O stretching mode). The #1 (and #4) conformer, which contains an intramolecular hydrogen bond pointing to the CO group, has a negative sign shifted to the low-energy region. In contrast, #3 (and #5) has a positive sign shifted to a higher energy region. These findings suggest that these two groups of conformers are responsible for the bisignate signal in the νCO region. These findings are consistent with the temperature-dependent IR/VCD spectra, which indicate that a decrease in temperature leads to an increase in the population of the most stable structure (#1).

**Figure 7:**
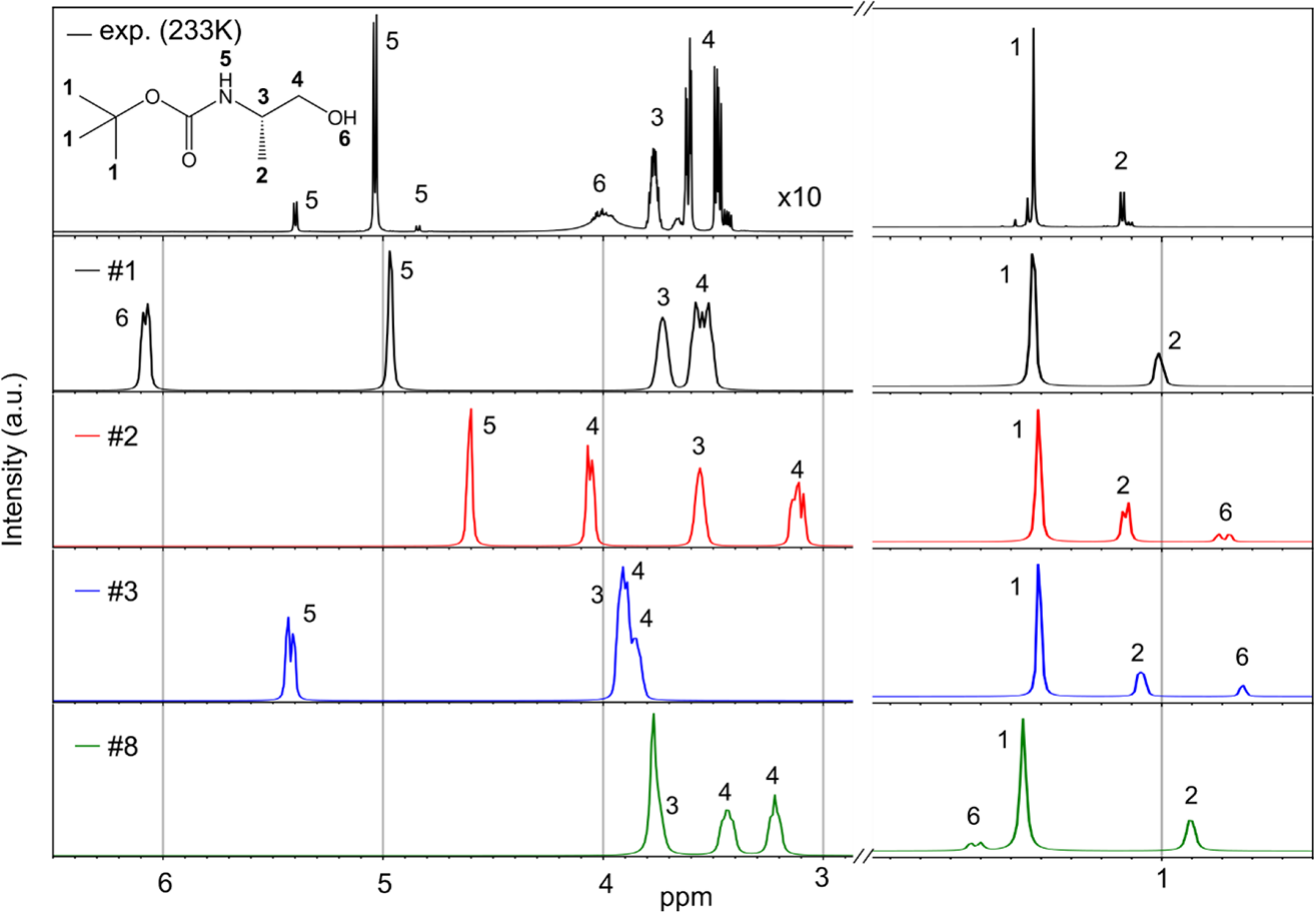
Comparison between experimental and simulated NMR spectra. The experimental NMR spectrum shown was measured at 233 K. The simulated NMR spectrum of the conformers #1, 2, 3, and 8 is shown as obtained without scaling.

Remarkably, the simulation results also show excellent agreement with the experimental NMR spectra. While the IR bands for νNH (3445 cm^–1^) were insensitive to conformational differences, the amide proton signals of NMR showed high sensitivity. The intense doublet centered at 5.04 ppm in the experimental data (Figure 7 top, Figure 2D—233 K for the zoom) can be assigned to the amide proton of the conformer #1 (4.97 ppm). Then, the doublet centered at 4.84 and 5.40 ppm in the experiment can be assigned to the amide proton of the conformers #2 (4.60 ppm) and #3 (5.42 ppm), respectively. Although the amide proton of the conformer #8 (the first *cis* conformer in the energy landscape) appears at 3.77 ppm in the simulation, no additional doublet peak could be observed around this chemical shift region. The broad peak centered at 4.00 ppm is assigned as the OH proton. This peak shows the highest downfield shift when the temperature decreases (Figure 2D). The chemical shift of the OH proton shows high sensitivity to each conformer in the simulation. Unfortunately, labile character of the OH makes it difficult to use it as a marker of each conformer due to efficient proton exchange with every labile group in the structure (e.g., NH) and water traces from the environment. We verified that the experimental result for IR and VCD was not affected by the presence of a small number of water molecules by measuring temperature dependent spectrum of the chloroform (Figure S12). The complete assignment of NMR peaks from simulations can be found in Figure S13. Compared with the other carbamate systems explored before,^72,73^ the lowest energy *cis* conformer is found at only 1.27 kcal/mol above the global minimum in this system. Yet, it was challenging to experimentally confirm its presence in solution based on vibrational and NMR spectroscopy used in this study.

Our results demonstrate that the use of HLT calculations provides an accurate description of the experimental findings. However, the scalability of this approach is limited, as the computational cost increases significantly with the size of the system being explored. Therefore, to further investigate sequence-defined oligocarbamates, it is crucial to evaluate whether a comparable level of accuracy can be achieved using the LLT. In the following analysis, we will compare the performance of LLT and HLT to assess the feasibility of using LLT for future investigations.

**Figure 8:**
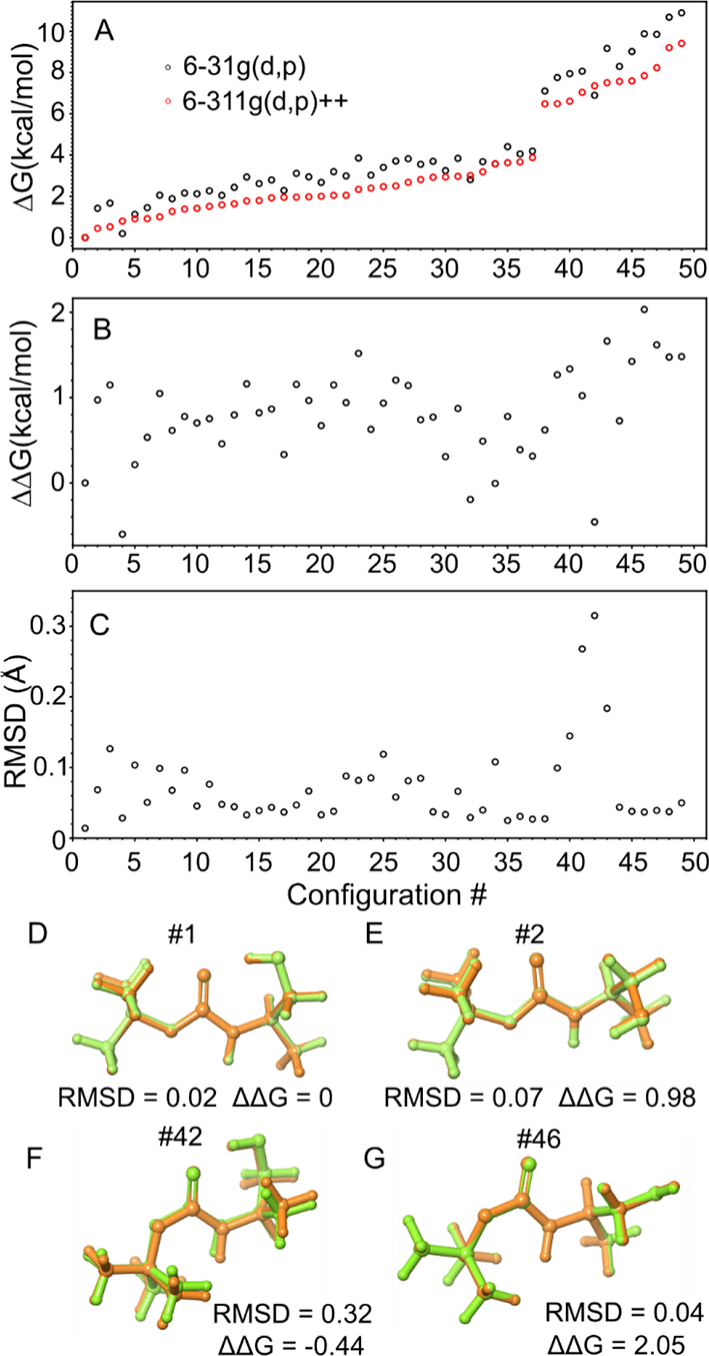
(A) Relative Gibbs free energy profiles for the 49 optimized geometries using LLT (black open circle) and HLT (red open circle). Values were ranked in ascending order based on Δ*G* calculated by HLT. (B) The ΔΔ*G* (ΔΔ*G* = Δ*G*_HLT_ – Δ*G*_LLT_) for each configuration. (C) Root-mean-square differences (RMSD) between the structure calculated by LLT and HLT for each conformer. (D, E) Geometry overlay for the #1 and #2 structures optimized at LLT (green) and HLT (orange). (F, G) Geometry overlay for the #42 (the highest RMSD) and #46 structures (the highest ΔΔ*G*) optimized at LLT (green) and HLT (orange).

Figure 8A compares the relative Gibbs free energy profile obtained from optimizing the geometry using both LLT and HLT theoretical calculations. The ranking of HLT (Figure 3) was used for comparison. The LLT approach predicted the global minimum and overall trend of the Gibbs free energy profile, as seen in Figure 8A. However, some differences are observed between the two methods, as shown in panel B (|ΔΔ*G*| <2 kcal mol^–1^). These discrepancies are significant enough to raise cautions about the Boltzmann factors obtained using LLT. Nevertheless, the optimized geometries obtained using LLT were found to agree perfectly with those obtained using HLT, as indicated by the low root-mean-square deviation (RMSD) value (RMSD <0.3 Å, Figure 8C). For example, Figure 8D,E presents an overlay of the optimized structures obtained using LLT and HLT for conformers #1 and #2, respectively. Figure 8F,G shows the conformer with the highest RMSD value (#42) and that with the highest ΔΔ*G* value, respectively. Furthermore, Figures S8 and S9 compare the shape of the molecular orbitals and the atomic charge distribution together with the electric dipole moment of selected structures obtained in the two LTs. In this sense, LLT catch the main features of the HLT.

Finally, we compared the simulated IR and VCD spectra obtained from the two levels of theory (LLT and HLT). We used cosine similarity (Sc) as a descriptor to evaluate the similarity between spectra. This approach treats the simulated spectrum at LLT and HLT as vectors in an inner product space. The cosine similarity is defined as the cosine of the angle between the two vectors, i.e., the dot product of the vectors divided by their norms. Therefore, this descriptor always belongs to the interval [−1,1]. Extreme values of Sc, i.e., Sc = 1 or Sc = −1, indicate that the spectra are proportional or opposite, while Sc = 0 indicates that the two spectra are orthogonal. For the IR spectra, the cosine similarity values were neatly bounded within the interval [0,1].

**Figure 9:**
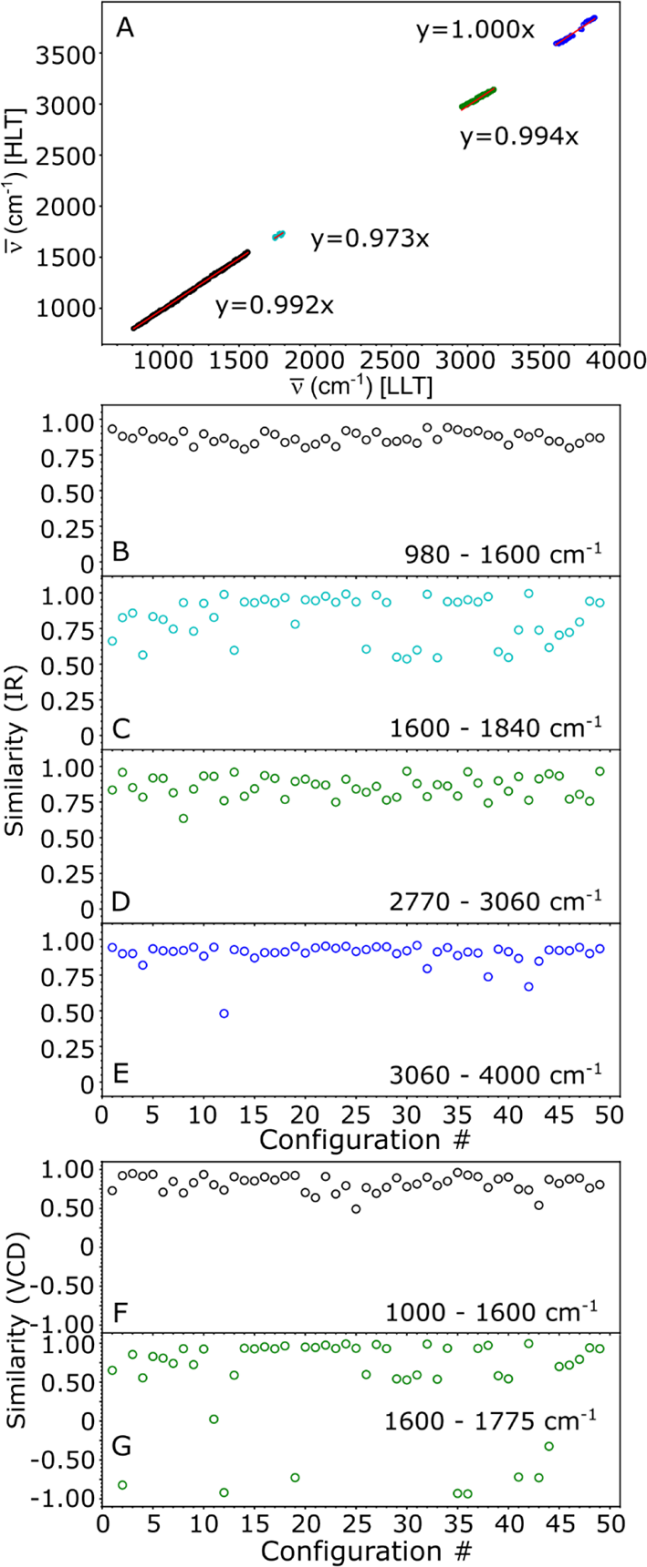
(A) Regression between the harmonic frequencies calculated at LLT and HLT. A linear regression (solid line) with zero intercept is applied depending on the region. The slopes are used as scaling factors during the cosine similarity calculations. See text for additional details. (B–E) The similarity analysis of the IR spectra spectrum between the two levels of theory. (F, G) The similarity analysis of the VCD spectra between the two levels of theory.

To accurately estimate Sc values for IR or VCD spectra obtained using LLT and HLT, it is necessary to correct for the shift in the position of harmonic frequencies that result from using different basis set sizes between the two methods. To accomplish this, we introduced a region-dependent scaling factor. Figure 9A displays a regression analysis of the harmonic frequencies computed at LLT with those calculated at HLT. Four regions were identified and fitted with a linear curve with a zero intercept. The resulting scaling factors are then applied to the harmonic frequencies of LLT spectra when estimating the similarity with HLT spectra.

Figure 9B–E presents Sc values for IR spectra obtained from LLT and HLT, grouped by region. The mean and standard deviation of Sc per region were calculated as follows: 0.87 ± 0.04 (980–1600 cm^–1^), 0.8 ± 0.2 (1600–1840 cm^–1^), 0.86 ± 0.07 (2770–3060 cm^–1^), and 0.90 ± 0.08 (3060–4000 cm^–1^). Overall, a high degree of similarity was observed between the two levels of theory, indicating that LLT can capture the behavior of HLT concerning IR spectra. The lowest Sc values were observed in the −CO stretching region, which is known to be highly sensitive to environmental changes. Regarding the VCD spectra, Figure 9F,G displays the Sc values obtained for LLT and HLT, again grouped by region. In the 980–1600 cm^–1^ region, high Sc values of 0.8 ± 0.1 were obtained. However, in the 1600–1775 cm^–1^ region, some Sc values were negative, indicating that the sign of the VCD is opposite between the two spectra. Figure S14 provide further information regarding the differences observed between LLT and HLT for these data points. These negative Sc values drastically reduced the mean and increased the standard deviation of Sc to 0.5 ± 0.6. While most of the results obtained using LLT are reliable, it is important to note that the VCD spectrum in the ν(CO) region requires special attention based on the results obtained in this study.

## Conclusions

We combined IR, VCD and NMR spectroscopy with DFT calculations to understand the conformation of Boc-carbamate monomer units in chloroform solution. Detailed analysis of simulated conformation revealed that carbamate units are plane, presumably due to the extended delocalization of π-electrons on the backbone. *Cis* configurations can be energetically stable for carbamates, while peptides are mostly found as *trans* configurations. The stabilization of the *cis* configurations could be also supported by delocalization of π-electrons on the backbone, and in some cases, the oxygen next to the amide bond worked as a hydrogen bonding acceptor to stabilize the *cis* configuration. Although it was challenging to experimentally show the presence of the *cis* conformer in this study, the theoretically identified *cis* conformer at relatively low energy and its abundance could play an important role in the single chain folding conformation of oligocarbamates based on this system. DFT calculations based on the Boc-carbamate monomer showed an excellent agreement with the experimental IR, VCD, and NMR spectra. Based on the comparison, we could assign several conformers that the Boc-carbamate monomer unit can assume in chloroform. We also demonstrated that the lower level theory was sufficient to reproduce the results obtained by the high level theory, which means that the oligocarbamate system of higher molar mass can be studied using the established methodology herein. This study lays an important foundation for future developments of carbamate-based sequence-defined polymer material design.

## Supporting Information Available

Additional IR, VCD, and ^1^HNMR spectra, 2D plot of the reduced density gradient and the electron density, molecular orbitals, the atomic charges and normal modes of conformers discussed in the text, and the comparison of the simulated IR and VCD spectrum between LLT and HLT (PDF)

## Notes

Data supporting the findings of this manuscript is available on Yareta (doi: 10.26037/yareta:zc3q5wi6zjbipfhpzt5afvrsne).
