# Supplementary material for: Unveiling the Configurational Landscape of Carbamate: Paving the Way for Designing Functional Sequence-Defined Polymers
Source: J Phys Chem A. 2023 Aug 25;127(35):7309–22. doi: 10.1021/acs.jpca.3c02442 (PMC10493977; doi:10.1021/acs.jpca.3c02442)
Supplement: Supplementary file 1 — jp3c02442_si_001.pdf [file jp3c02442_si_001.pdf]

Supporting information for

Unveiling the Configurational Landscape of Carbamate: Paving the Way for Designing Functional Sequence-Defined Polymers

Ariel F. Perez Mellor,[1, ‡,] Johanna Brazard, [1, ‡,]* Sara Kozub,[2] Thomas Bürgi,[1] Roza Szweda,[2] Takuji B. M. Adachi[1,]**

[1]Department of Physical Chemistry, Sciences II, University of Geneva, 30, Quai Ernest Ansermet, 1211 Geneva, Switzerland

[2] Łukasiewicz Research Network – PORT Polish Center for Technology Development

Stabłowicka 147, 54-066 Wrocław, Poland

Table of Contents:

Section SI1. Supplementary figures:

Figure S1. Raw VCD spectra and the baseline correction of two enantiomers of Boc-carbamates

Figure S2. ^{1}H shifts of Tetramethylsilane (TMS) and Chloroform.

Figure S3. IR spectrum of Boc-carbamate at three different concentration

Figure S4. Full range of the ^{1}H NMR spectrum of Boc-carbamate

Figure S5. 2D plot of the reduced density gradient (*s*) and the electron density (ρ) of the conformer #1-8.

Figure S6. Distribution of interatomic distances between each atom and H26 (The full data of Figure 4 in the main text).

Figure S7. The structural difference between the conformer #37 and #38

Figure S8. Molecular orbitals of the conformer #1, #2, and #8 calculated using LLT and HLT

Figure S9. The atomic charges computed from the atomic axial polar tensor (APT) of the eight most stable structures

Figure S10. The illustration of the normal mode displacement

Figure S11. IR and NMR spectra of two enantiomers of Boc-carbamate.

Figure S12. IR spectrum of Chloroform at different temperatures.

Figure S13. ^{1}H NMR simulation results and assignment of the peaks

Figure S14. The comparison of the simulated IR and VCD spectrum between the LLT and HLT.

Section SI2. Supplementary table:

Table S1. The additional information of the energy for the eight lowest conformers

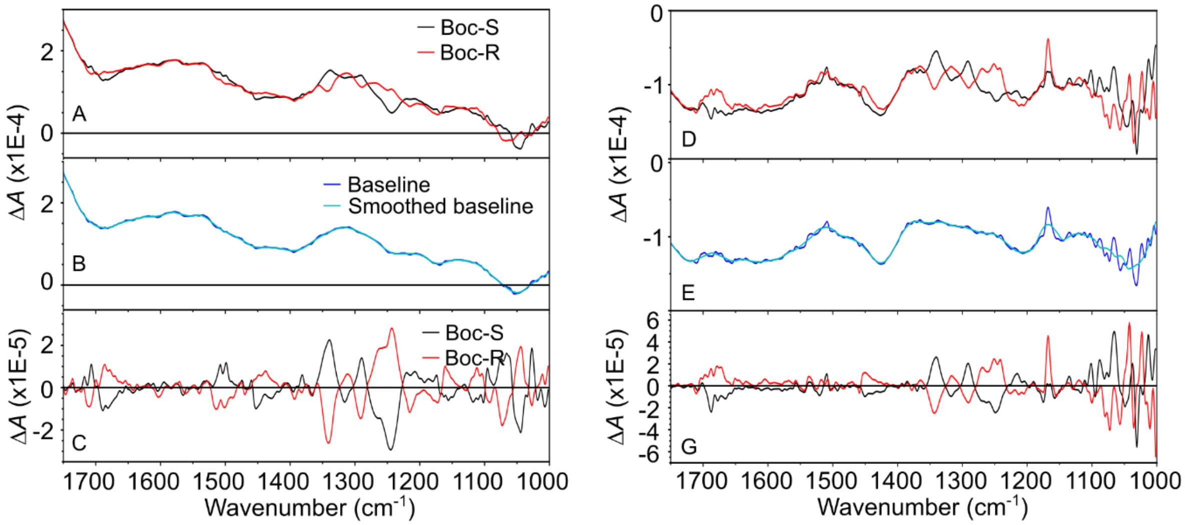

Figure S1. Comparative analysis of VCD spectra for enantiomers of Boc-Carbamates: Raw VCD spectra for two enantiomers of Boc-carbamates (A) at 300 K and (D) at 263 K. Calculation of the averaged VCD spectra obtained by combining half of the sum of the individual enantiomers' spectra (B) at 300 K and (E) at 263 K, serving as a baseline reference. Savitzky-Golay algorithm was used for smoothing the baseline (50 points window, and 2nd order polynomial). Baseline-corrected VCD spectra for both enantiomers of Boc-carbamates (C) at 300 K and (G) at 263 K.

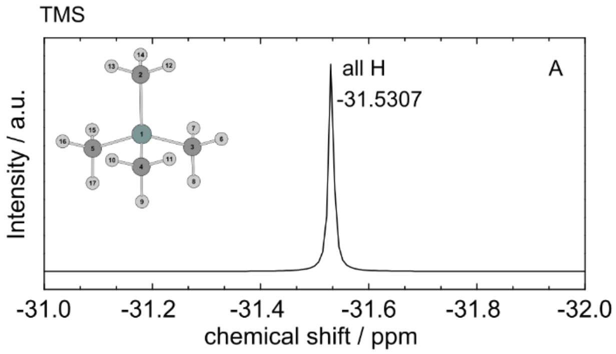

Figure S2. Simulated ^{1}H shifts of (A) Tetramethylsilane (TMS) and (B) Chloroform.

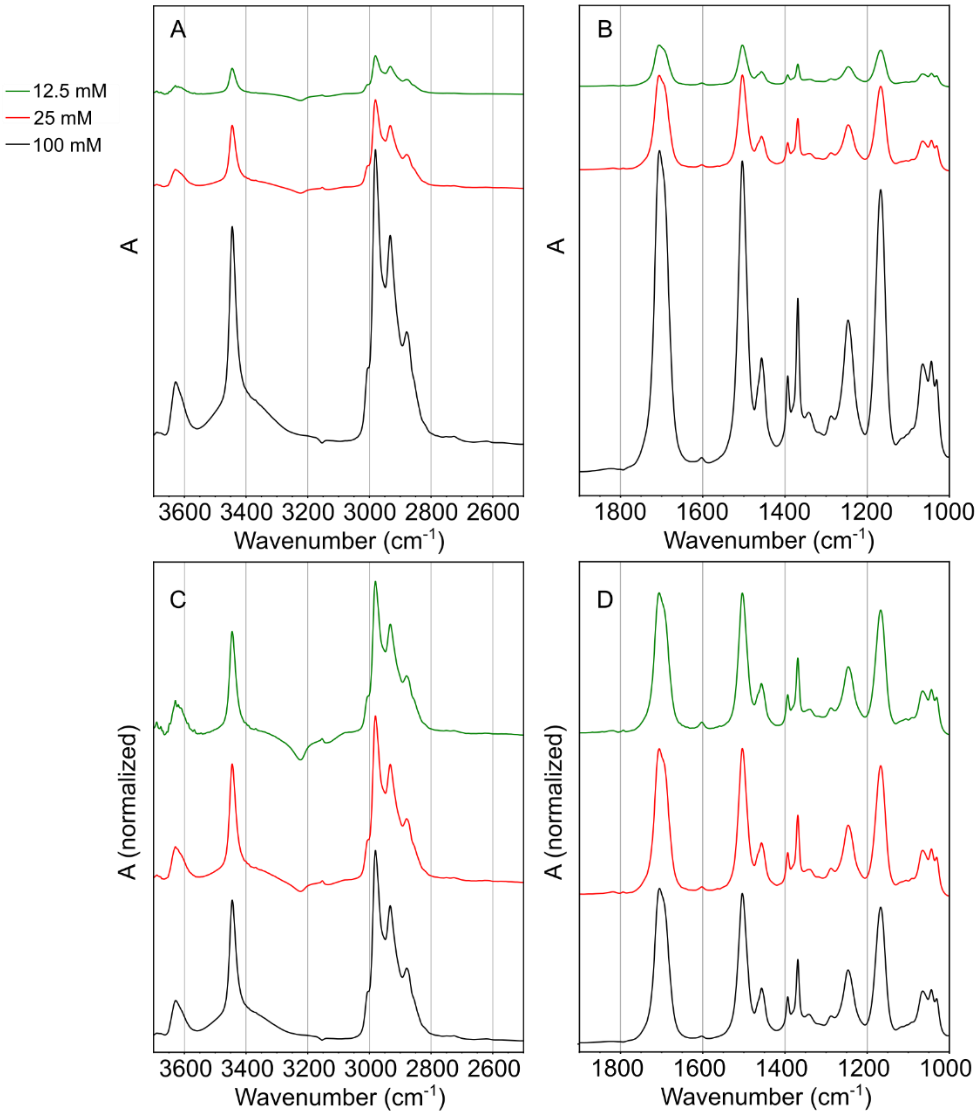

Figure S3. IR spectrum of Boc-carbamate at three different concentration (12.5, 25, and 100 mM). (A-B) raw data and (C-D) Normalized data.

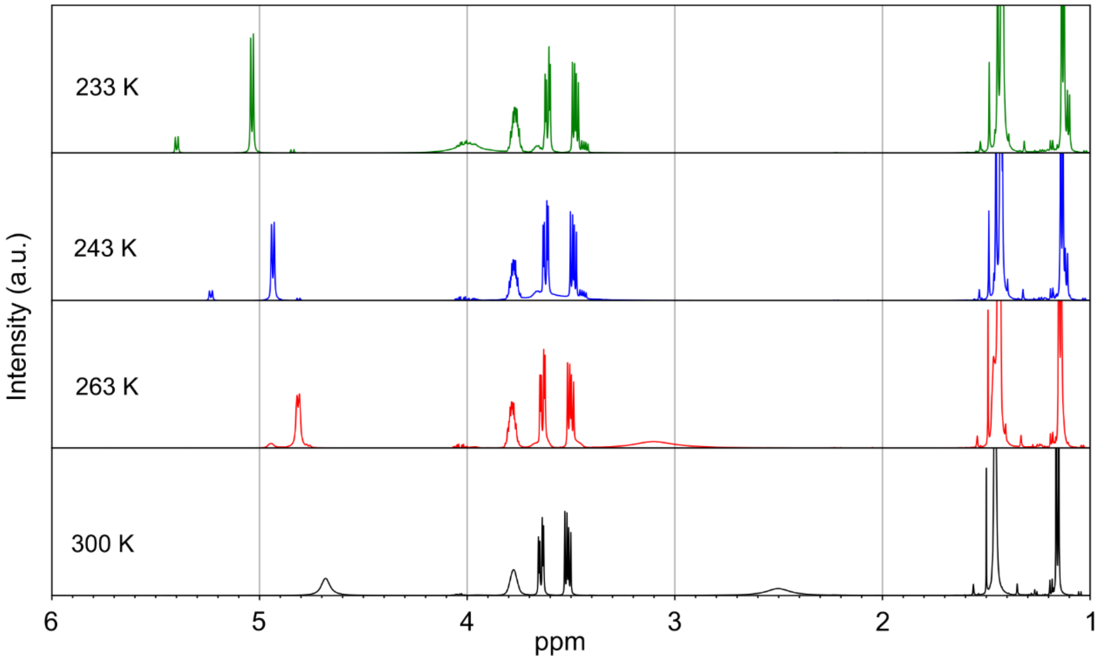

Figure S4. Full range of the NMR spectrum of Boc-carbamate monomer at room and low temperatures.

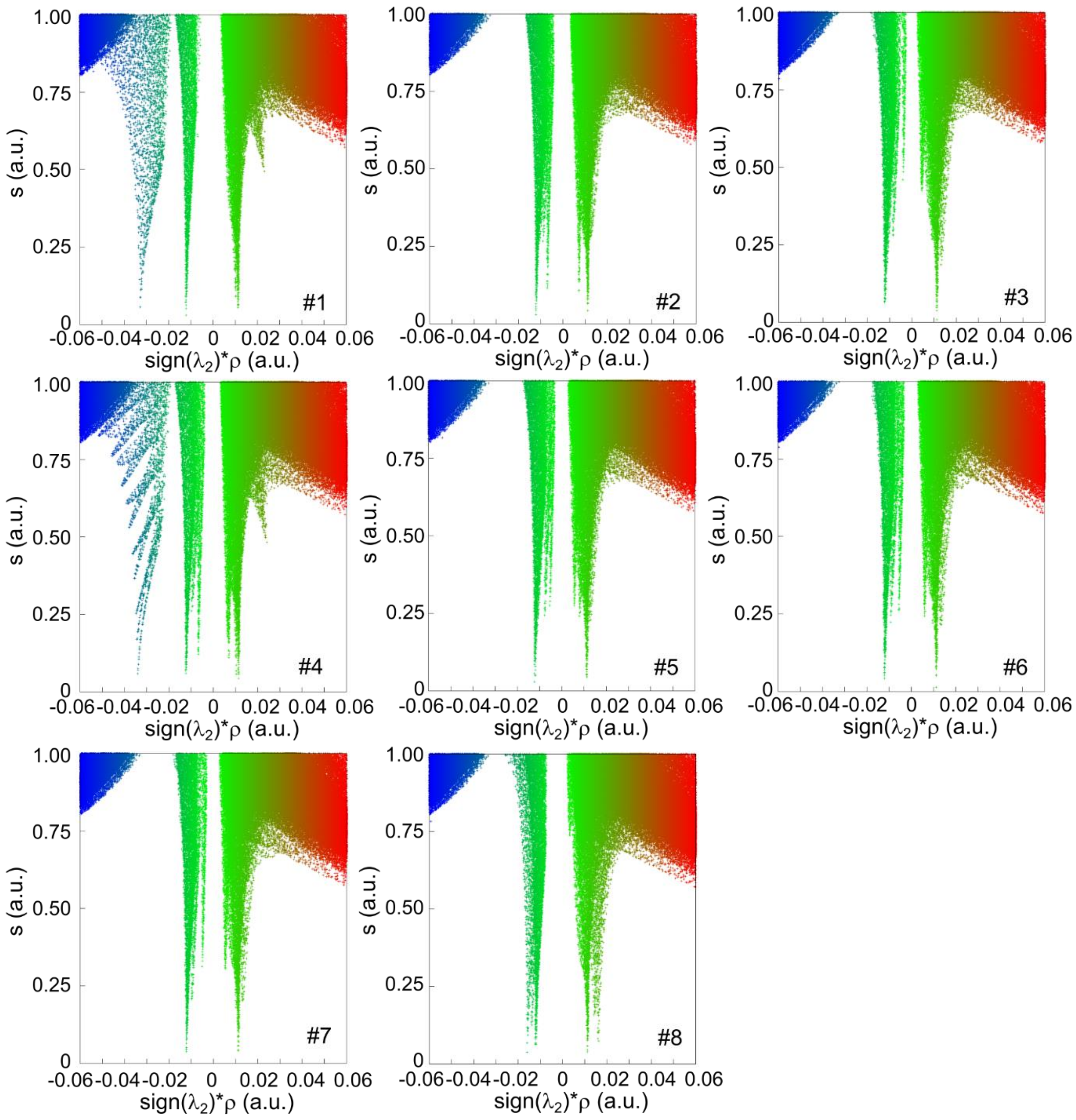

Figure S5. NCI plot of the eight lowest energy conformers.

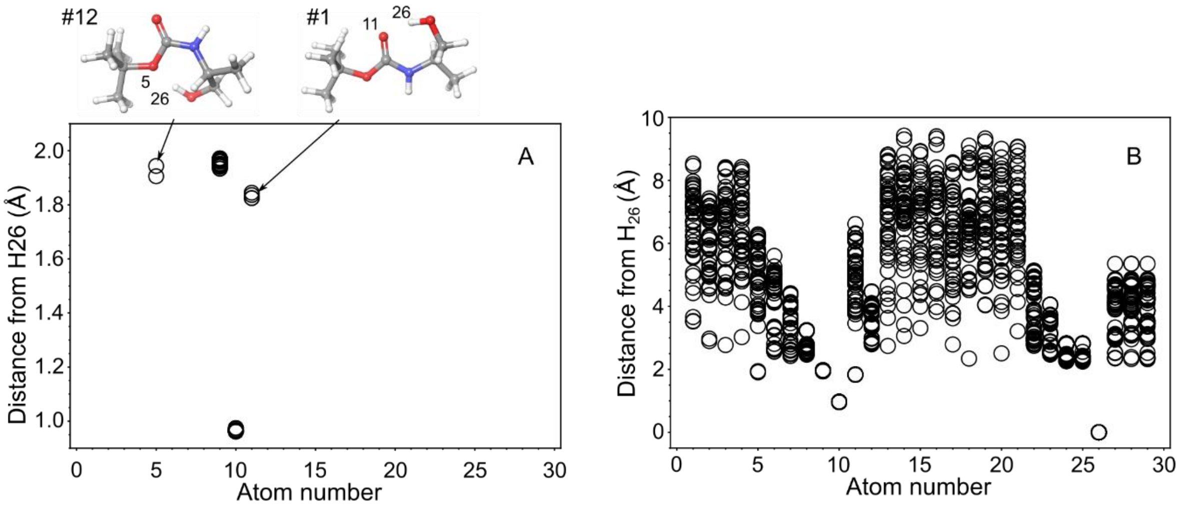

Figure S6. A) Distribution of interatomic distances for each atom (labeled in Figure 1) and H_{26}. Only the range for hydrogen bond and covalent bond distances (0.9 – 2.1 Å) are shown. The conformation of #1 is shown as an inset as a representative of the points at the atom number 11 (#1, #4, #38 and #42) and that of #12 as a representative of the points at the atom number 5 (#12, and #32). B) The full data set highlighted in the panel A.

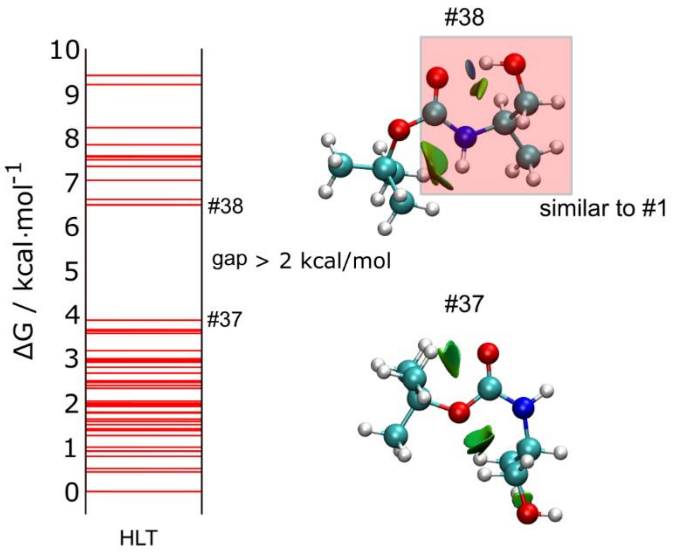

Figure S7. The free energy landscape of conformers which shows more than 2 kcal/mol energy gap between the conformer #37 and #38. The conformer #37 and #38 is shown on the right.

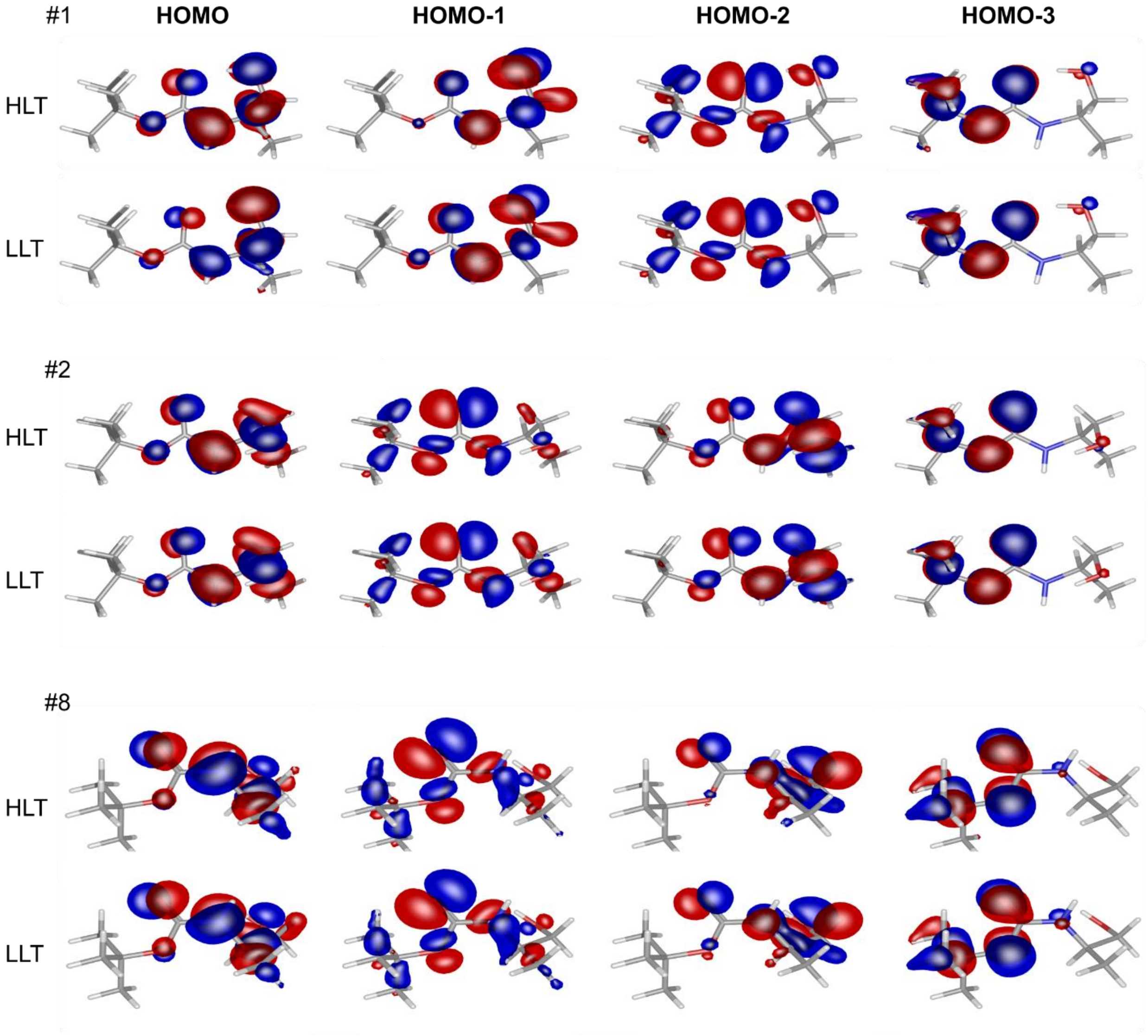

Figure S8. Molecular orbitals of the conformer #1, #2, and #8 calculated using LLT and HLT, which shows the delocalization of molecular orbitals. The isovalue is fixed at 0.04 a.u.

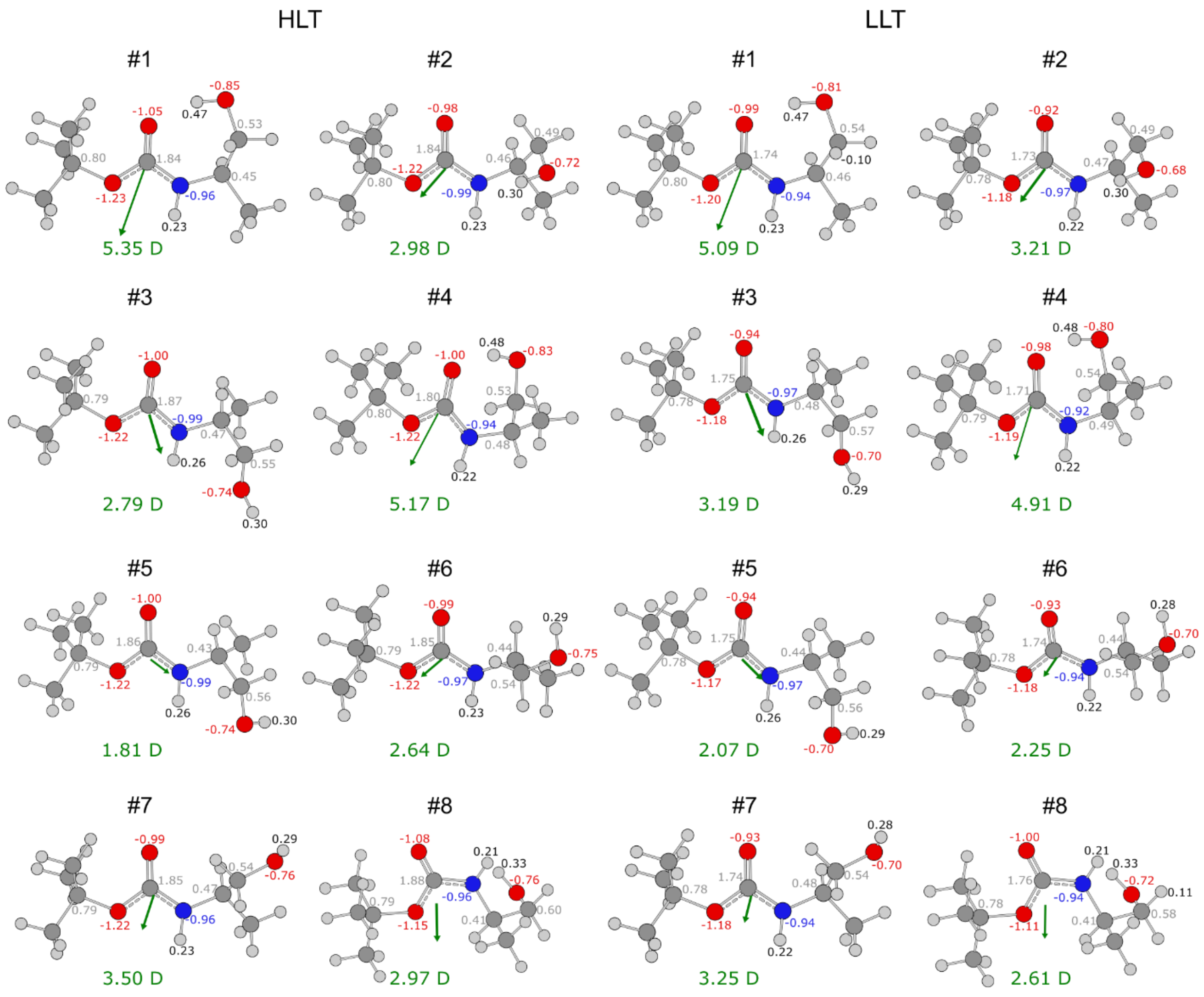

Figure S9. The atomic charges computed from the atomic axial polar tensor (APT) of the eight most stable structures together with the electric dipole moment. The result of LLT is similar to that of HLT, demonstrating the validity of LLT. Only atomic charges greater than 0.1 are shown.

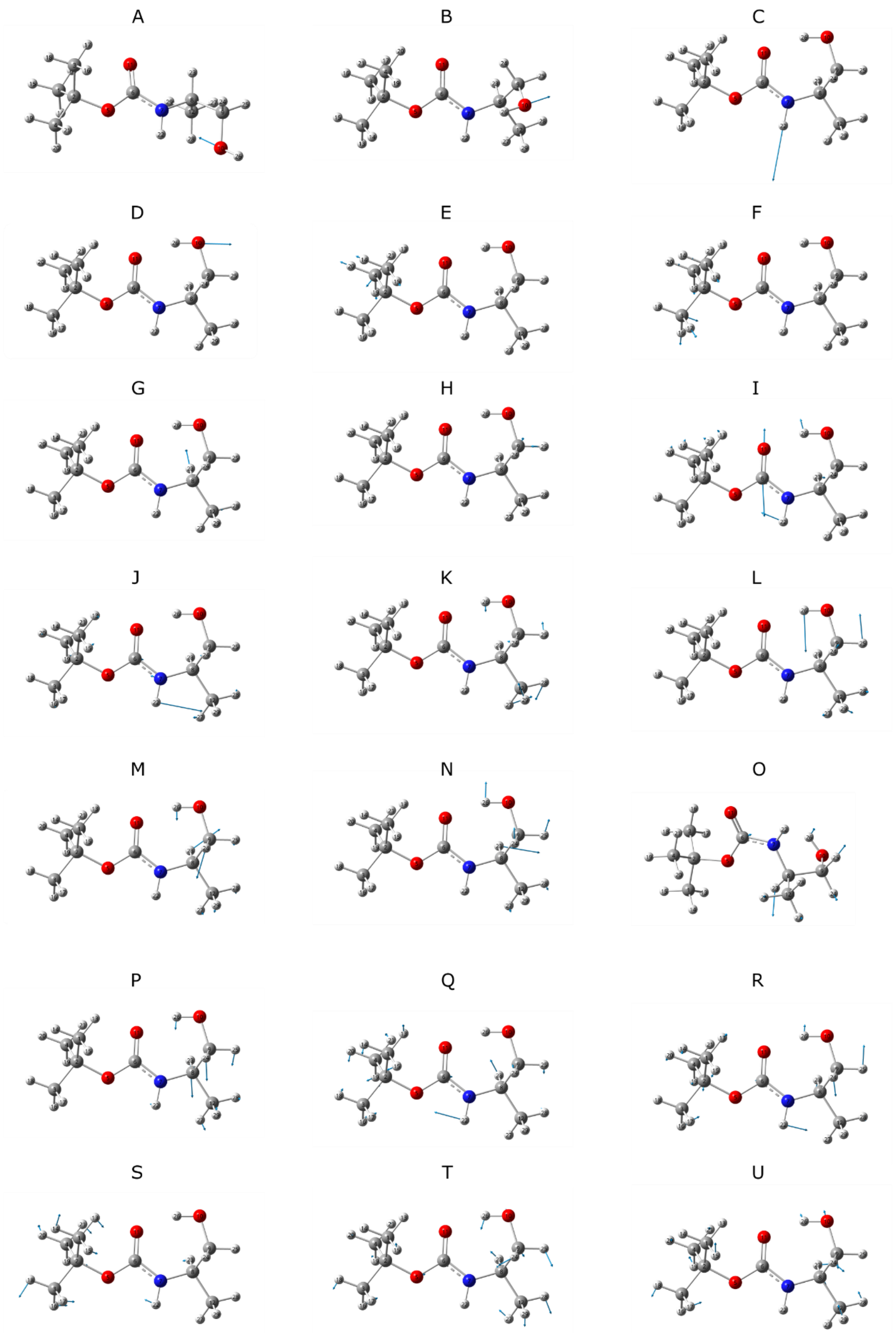

Figure S10. The illustration of the normal mode displacement referred in the Table 2 in the main text.

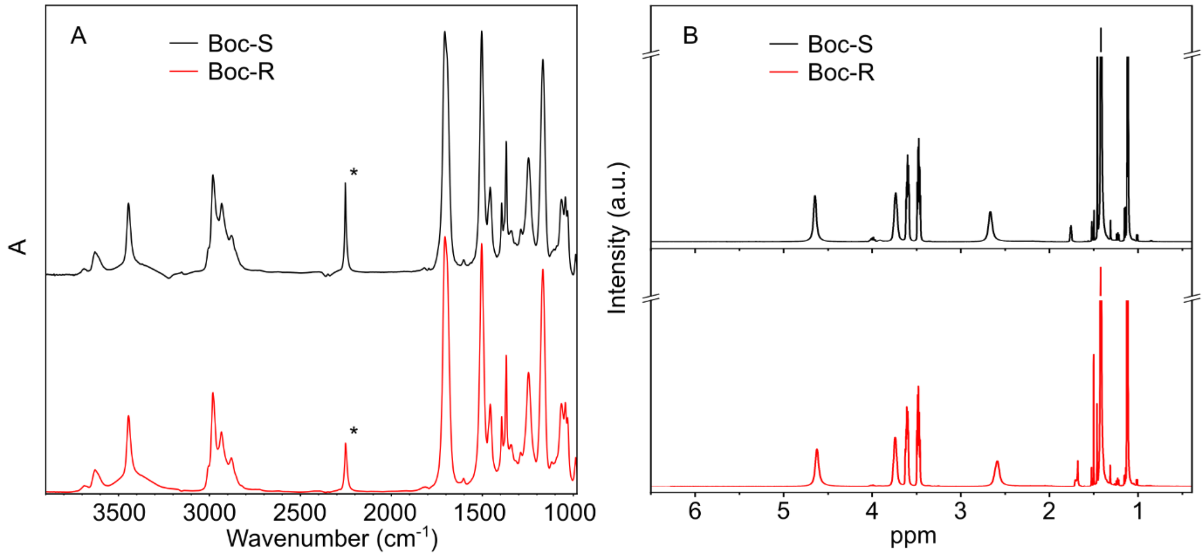

Figure S11. (A) IR and (B) NMR spectra of two enantiomers of Boc-carbamate. The peak denoted by * is due to the imperfect background subtraction of chloroform absorbance.

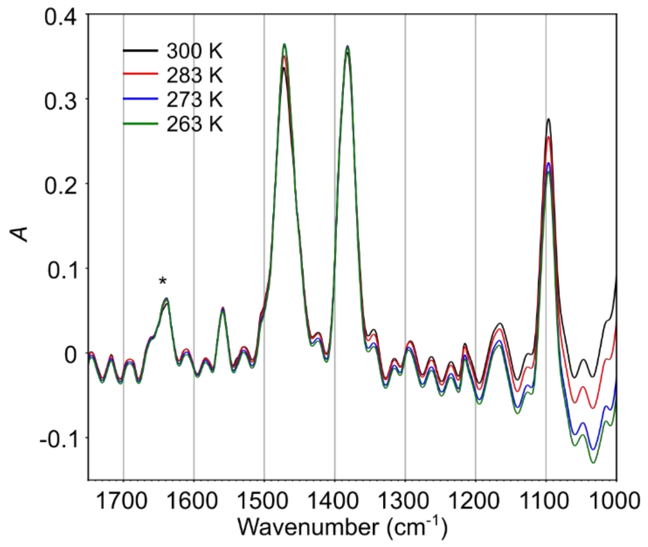

Figure S12. (A) IR spectrum of deuterated chloroform at different temperatures. * denotes the IR band of liquid water present in the solvent. Please note that this IR band does not change with varying temperature at the range used in our study.

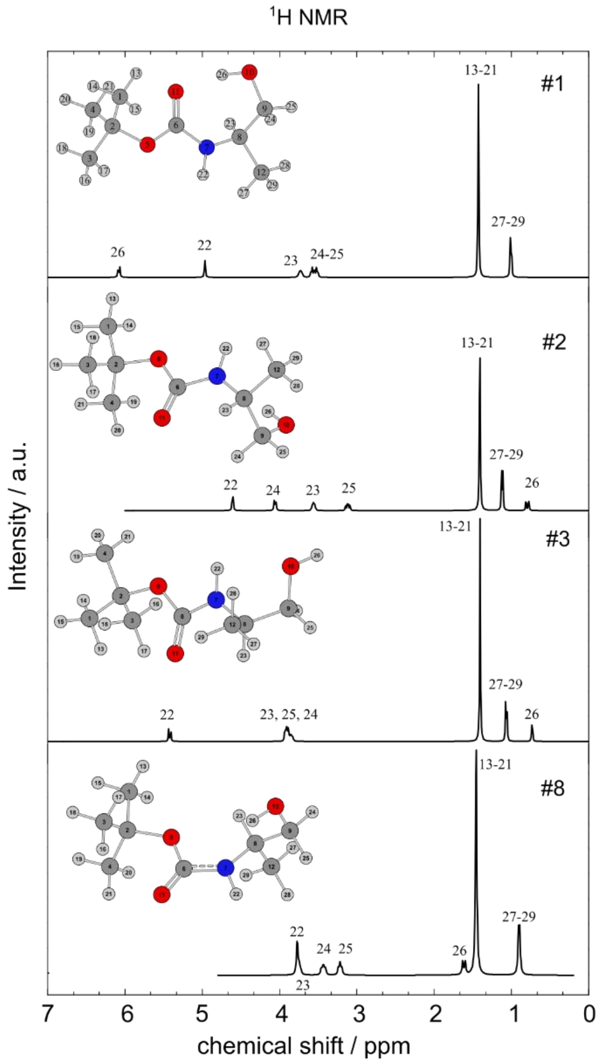

Figure S13. ^{1}H NMR simulation results and assignment of the peaks.

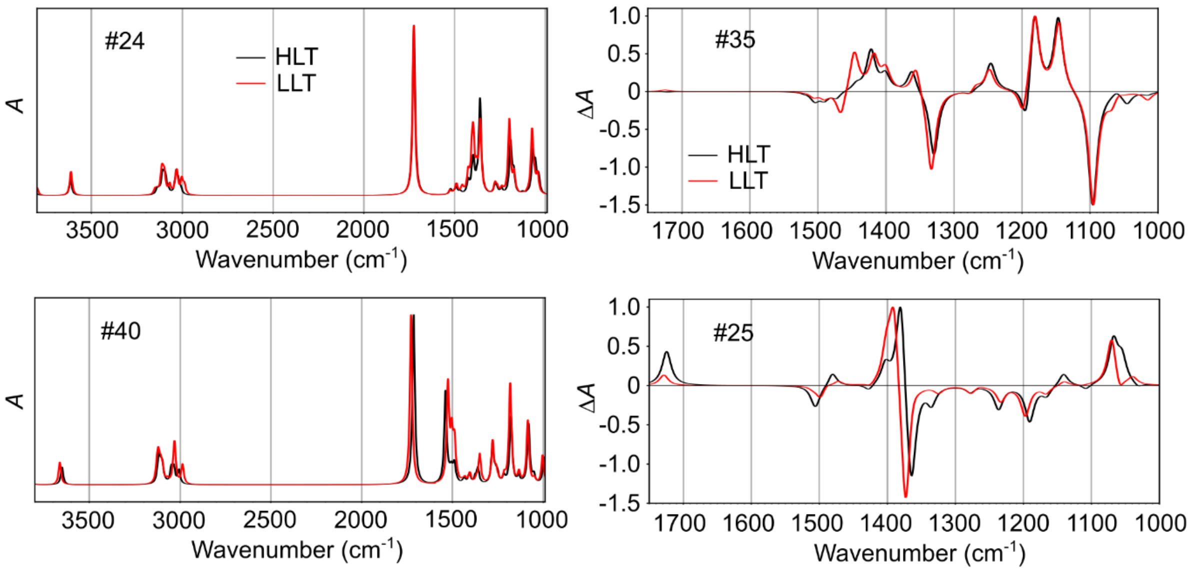

Figure S14. The comparison of the simulated IR and VCD spectrum between the LLT and HLT. For the comparison, the spectra calculated by LLT were corrected using the scaling factor described in the main text. The top row shows the spectral differences that gave the best similarity values and the bottom row shows the worst similarity values.

Table S1: The additional information of the energy for the eight lowest conformers

Conformation	ΔE + ZPE (kcal/mol)	Boltzmann weighted population (%)	ΔG (SATP) (kcal/mol)	Boltzmann weighted population (%)
#1	0.0	43	0.0	35
#2	0.79	11	0.45	16
#3	0.97	8.1	0.52	14
#4	0.45	20	0.80	9.0
#5	1.0	7.4	0.91	7.4
#6	1.3	4.7	0.92	7.3
#7	1.6	2.6	1.0	6.3
#8	1.4	3.7	1.3	4.0
